# Soluble PD-L1 works as a decoy in lung cancer immunotherapy via alternative polyadenylation

**DOI:** 10.1172/jci.insight.153323

**Published:** 2022-01-11

**Authors:** Ray Sagawa, Seiji Sakata, Bo Gong, Yosuke Seto, Ai Takemoto, Satoshi Takagi, Hironori Ninomiya, Noriko Yanagitani, Masayuki Nakao, Mingyon Mun, Ken Uchibori, Makoto Nishio, Yasunari Miyazaki, Yuichi Shiraishi, Seishi Ogawa, Keisuke Kataoka, Naoya Fujita, Kengo Takeuchi, Ryohei Katayama

**Affiliations:** 1 Division of Experimental Chemotherapy, Cancer Chemotherapy Center, Japanese Foundation for Cancer Research (JFCR), Ariake, Koto-ku, Tokyo, Japan.; 2 Department of Respiratory Medicine, Tokyo Medical and Dental University, Yushima, Bunkyo-ku, Tokyo, Japan.; 3 Pathology Project for Molecular Targets, Cancer Institute,; 4 Division of Pathology, Cancer Institute,; 5 Department of Thoracic Medical Oncology, the Cancer Institute Hospital, and; 6 Division of Thoracic Surgery, Cancer Institute, JFCR, Ariake, Koto-ku, Tokyo, Japan.; 7 Division of Genome Analysis Platform Development, National Cancer Center Research Institute, Tsukiji, Chuo-ku, Tokyo, Japan.; 8 Department of Pathology and Tumor Biology, Graduate School of Medicine, and; 9 Institute for the Advanced Study of Human Biology (WPI-ASHBi), Kyoto University, Yoshida-Konoe-cho, Sakyo-ku, Kyoto, Japan.; 10 Department of Molecular Hematology, Karolinska Instisute, Solnavägen, Solna, Stockholm, Sweden.; 11 Division of Hematology, Department of Medicine, Keio University School of Medicine, Shinanomachi, Shinjku-ku, Tokyo, Japan.; 12 Division of Molecular Oncology, National Cancer Center Research Institute, Tsukiji, Chuo-ku, Tokyo, Japan.; 13 Cancer Chemotherapy Center, JFCR, Ariake, Koto-ku, Tokyo, Japan.

**Keywords:** Immunology, Oncology, Immunotherapy, Lung cancer

## Abstract

Immune checkpoint therapy targeting the PD-1/PD-L1 axis is a potentially novel development in anticancer therapy and has been applied to clinical medicine. However, there are still some problems, including a relatively low response rate, innate mechanisms of resistance against immune checkpoint blockades, and the absence of reliable biomarkers to predict responsiveness. In this study of in vitro and in vivo models, we demonstrate that PD-L1–vInt4, a splicing variant of PD-L1, plays a role as a decoy in anti–PD-L1 antibody treatment. First, we showed that PD-L1–vInt4 was detectable in clinical samples and that it was possible to visualize the secreting variants with IHC. By overexpressing the PD-L1–secreted splicing variant on MC38 cells, we observed that an immune-suppressing effect was not induced by their secretion alone. We then demonstrated that PD-L1–vInt4 secretion resisted anti–PD-L1 antibody treatment, compared with WT PD-L1, which was explicable by the PD-L1–vInt4’s decoying of the anti–PD-L1 antibody. The decoying function of PD-L1 splicing variants may be one of the reasons for cancers being resistant to anti–PD-L1 therapy. Measuring serum PD-L1 levels might be helpful in deciding the therapeutic strategy.

## Graphical abstract

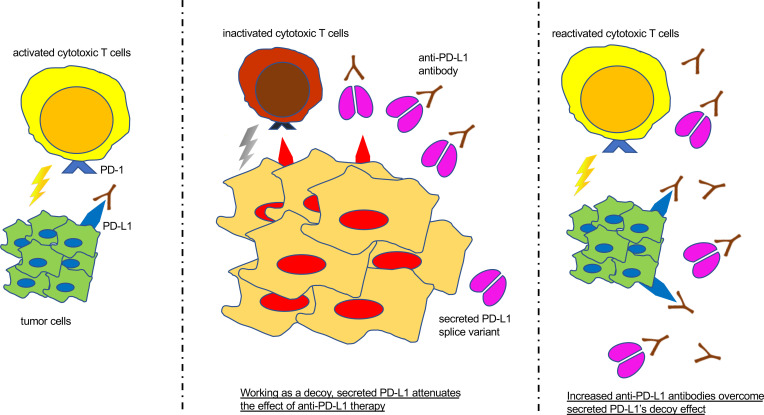

## Introduction

Cancer has long been a major leading cause of death. Starting from cytotoxic chemotherapy agents (e.g., cisplatin), different anticancer strategies have been devised by scientists. In particular, remarkable progress has been made with molecular targeted therapies, such as gefitinib (1) and cetuximab (2). The most recent strategy is immune checkpoint therapy; research in this area was awarded the Nobel Prize in Physiology or Medicine in 2018 (3–7). Immune checkpoint therapy inhibits immunosuppressive molecules, deactivating cytotoxic activities. It includes the PD-1/PD-L1 axis (6, 8, 9) and other immune checkpoints, such as cytotoxic T-lymphocyte–associated protein 4 (CTLA-4) (10) and lymphocyte activation gene 3 (LAG3) (11); these molecules have been shown to be possible, promising, and hopeful targets for anticancer therapy. There is increasing evidence that anti–PD-1/PD-L1 therapy is effective against malignant neoplasms, such as lung cancer (12, 13), melanoma (13, 14), Hodgkin’s lymphoma (15, 16), and renal cell carcinoma (13, 17). It has efficacy when used alone and possible additional and/or synergistic efficacy when used in combination therapy with other antitumoral agents (16). However, the efficacy of immune checkpoint inhibitor (ICI) monotherapy has been reported to be no more than 20%–32% (18, 19). Factors such as differences in patient background, tumor microenvironment (TME), and cancer type may adversely affect its therapeutic sensitivity; unfortunately, no absolute or versatile regimen is currently advocated. Some factors are reported to be useful in predicting the efficacy of immunotherapies, such as tumor mutational burden (20) and microsatellite instability (21). However, we cannot make use of them currently as authentic or trustworthy biomarkers.

PD-L1 (also known as CD274 or B7-H1) is a member of the B7 family and a well-known ligand of PD-1. It works as an immune checkpoint molecule to suppress cytotoxic T cells by forming microclusters containing T cell receptors (TCRs) and their costimulatory receptor CD28 (22) via recruitment of Src homology 2 domain–containing tyrosine phosphatase 2, which inhibits TCR signaling (23). It has been reported that PD-L1 is expressed on the surface of various malignant cancer cells and participates in immune escape from cytotoxic T cells through interaction with PD-1, which leads to their inactivation and exhaustion or impedes their proliferation (7, 24, 25), contributing to diseases including cancer and chronic infection. PD-L1 is known to have several variants other than the full-length protein, which contains extracellular, transmembrane, and cytoplasmic domains (26). It has been previously reported that structural alteration of PD-L1, through disruption of the 3′-untranslated region, had led to its aberrant expression in various types of cancers, including adult T cell leukemia and diffuse large B cell lymphoma (27). We previously reported that PD-L1–v242, a PD-L1 splice variant that does not possess a transmembrane domain and therefore exists in a soluble form, showed an immunosuppressive effect by working as a decoy for the anti–PD-L1 antibody (28).

Another splice variant, PD-L1–vInt4, has an amino acid sequence that is paused at certain locations of intron 4 of PD-L1; it is reported to show an immunosuppressive effect when dimerized by disulfide linkage via the cysteine contained in the tail-like structure of the spliced fourth intron (29). This variant is believed to be produced as a result of alternative polyadenylation in intron 4 immediately after exon 4 (27). Since the transmembrane domain of PD-L1 locates in the fifth exon, PD-L1–vInt4 lacks it and therefore exists as a soluble secreting variant.

Anti–PD-L1 therapy with chemotherapy has been one of the standard therapies in non–small cell lung cancer. In this study, we examined the protein and mRNA expression of PD-L1 variants. Surgically resected tumor and adjacent normal tissues were obtained to analyze PD-L1 expression, using an original design for amplicon sequencing and RNA-Seq. PD-L1 protein expression was evaluated by IHC with several anti–PD-L1 antibodies that recognize different epitopes of PD-L1: intracellular SP142 and extracellular E1J2J and 22C3. The PD-L1–v242 variant can be observed even in tumor tissues naive to ICI treatment, while PD-L1–vInt4 mRNA expression can be conjectured by RNA-Seq. IHC staining showed an interesting difference in some samples when the samples were stained with antibodies recognizing different domains: intracellular and extracellular. Analysis of the Cancer Genome Atlas (TCGA) data set revealed that some cancer types, such as diffuse large B cell lymphoma (DLBCL), thymoma, and lung squamous cell carcinoma (LUSC) frequently expressed PD-L1–vInt4. To examine whether PD-L1–vInt4 works as a decoy for anti–PD-L1 antibody to suppress or attenuate the efficacy of anti–PD-L1 therapy, we designed and performed in vitro assays and in vivo experiments inoculating MC38 cells expressing PD-L1 variants into C57BL/6 mice; we then treated them with anti–PD-L1 antibodies. The results suggested that PD-L1–vInt4 has a certain effect on anti–PD-L1 therapy.

## Results

### Evaluation of mRNA and protein expression of PD-L1 variants in LUSC.

**Figure 1:**
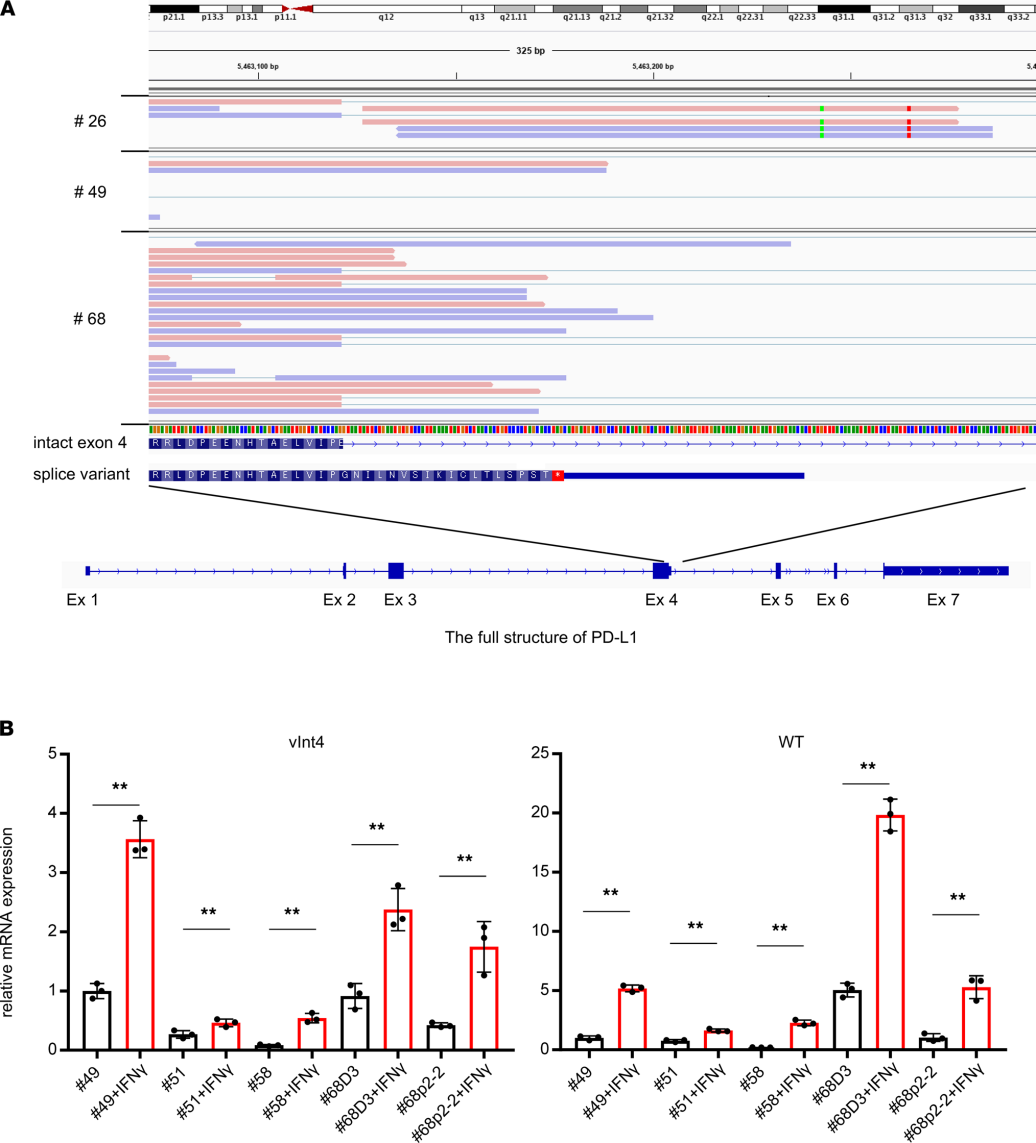
Splicing variant PD-L1–vInt4 mRNA was detected in LUSC clinical specimens and induced with IFN-γ treatment. (**A**) mRNA of PD-L1–vInt4 was detected in clinical samples by RNA-Seq. Additional and aberrant sequences are seen in the intron 4 domain in the structure map. (**B**) mRNA expression in clinical cell lines with or without IFN-γ treatment evaluated with quantitative PCR. Black bars represent naive cells, and red bars represent IFN-γ treatment. Data represent mean ± SEM. **P < 0.01 by paired 2-tail Student’s *t* test.

**Figure 2:**
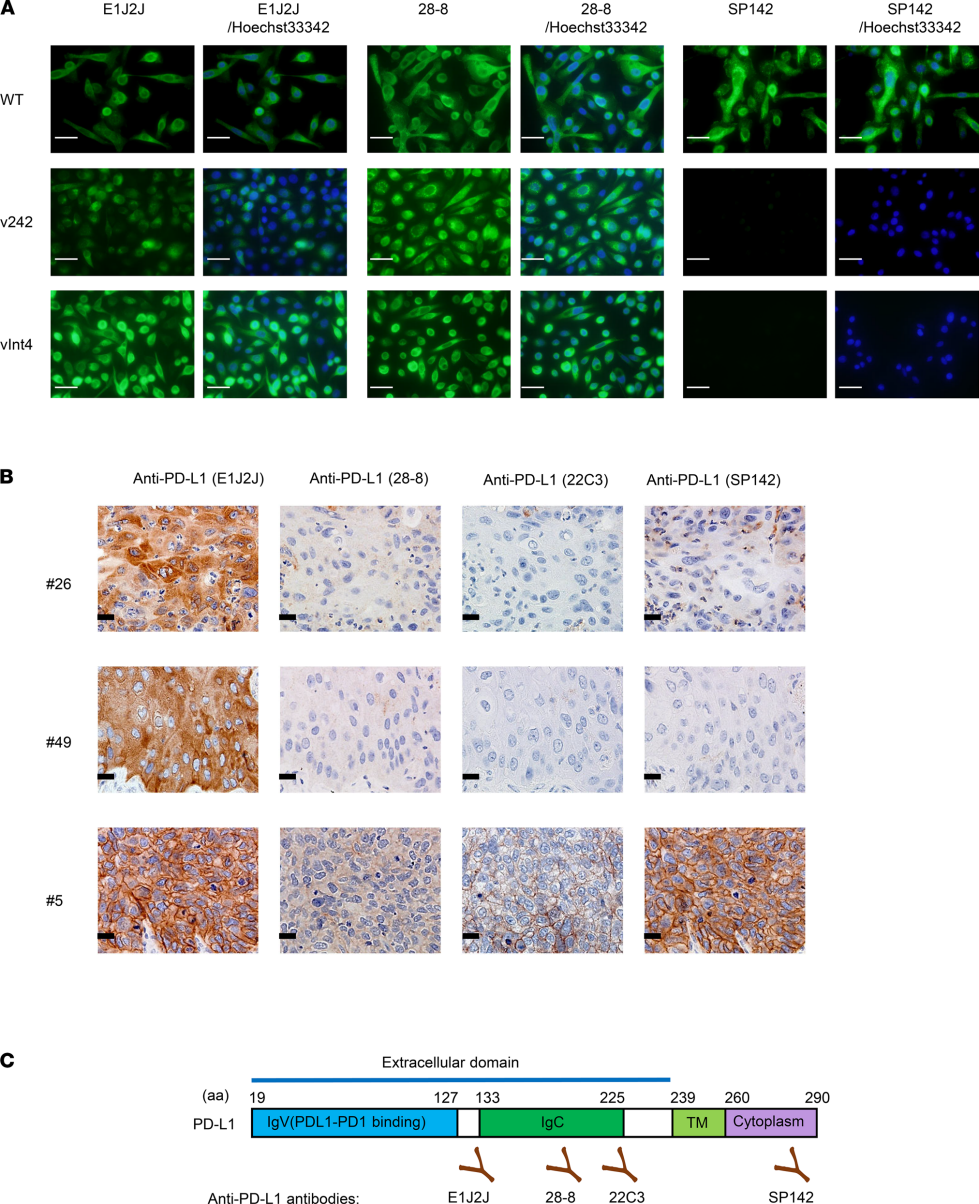
Secreted PD-L1 variants were not stainable with SP142 (the intracellular domain–recognizing antibody), which shows that they can be differentiated with IHC in clinical specimens. (**A**) Immunofluorescence staining of PD-L1 expressed in PC9 cells. The nuclei of the cells are stained with Hoechst 33342. This shows that secreted variants are detectable by comparing staining patterns, with 2 or more different antibodies recognizing different epitopes. Scale bars: 20 μm. The exposure time was 40 ms for 28-8 and 60 ms for E1J2J. (**B**) Secreted PD-L1 variants were visually identifiable in IHC. Scale bars: 20 μm. The numbers represent each case number, in accordance with Table 1. A PD-L1 WT–expressed case #5 is shown as a positive control for SP142. (**C**) The structure of full-length PD-L1 and recognition sites of each antibody.

In our previous study, we identified several PD-L1 variants (PD-L1–v242 and –v229) in samples from anti–PD-L1–treated lung cancer patients (LUSC and *EGFR-*mutated lung adenocarcinoma) (28). We investigated the staining pattern of PD-L1 in IHC with 2 different antibodies in 52 surgically obtained specimens of LUSC with paired adjacent normal lung (Table 1). PD-L1 was PCR-amplified from cDNA of these samples and sequenced with next-generation sequencing using the Nextera XT library preparation kit (Illumina), and it revealed that some of the samples expressed PD-L1 splice variants (Supplemental Figures 1 and 2; supplemental material available online with this article; https://doi.org/10.1172/jci.insight.153323DS1). In addition, the PD-L1–vInt4 variant harboring alternative poly A after exon 4 was detected by RNA-Seq data in several LUSC specimens (Figure 1A). We evaluated the protein expression pattern with immunofluorescence staining and IHC staining using 3 different anti–PD-L1 antibodies, recognizing distinct epitopes and several other antibodies, such as anti–β-2 microglobulin (β2M), HLA, and CD8 (Supplemental Figure 3). An SP142 antibody known to bind to the intracellular domain of PD-L1 clearly stained full-length PD-L1 expressed in PC9 cells, but the staining fell off when it was applied to secreted variants overexpressed in PC9 cells (Figure 2A). From the IHC staining of formalin-fixed, paraffin-embedded (FFPE) LUSC surgical tissues, we found several cases where PD-L1 was not detectable with SP142, though it was incontrovertibly positive with E1J2J. When we investigated the RNA-Seq data, PD-L1–vInt4 was detected in some of the cases (Figure 1A, Figure 2B, and Supplemental Figures 1 and 2). PD-L1–vInt4 was also inducible with IFN-γ treatment in some of the cell lines derived from our clinical samples (Figure 1B). As shown in the figures, the cytoplasm of the tumor cells was diffusely stained, as were the membranes, in the samples that included secreted PD-L1 variants when we used E1J2J (Figure 2B and Table 1), an antibody that binds to the closer site of the N-terminus extracellular domain (Figure 2C). By contrast, the staining of the cytoplasm fell off when using antibody SP142 (Figure 2B, Table 1, and Supplemental Figure 1), which recognizes the C-terminus intracellular domain (Figure 2C). It is noteworthy that several LUSC samples were negative for β2M and HLA, which suggests that those tumors might escape from tumor-recognizing immune cells by losing antigen presentation through loss of HLA expression (Table 1 and Supplemental Figure 3). Since tumor tissue contains nontumor cells with high expression of PD-L1, such as macrophages and DCs, it might be difficult to evaluate the ratio of secreted PD-L1 expression by RNA-Seq or amplicon sequencing from bulk tumor tissue. However, IHC data with at least 2 antibodies recognizing extracellular and intracellular domains can predict the expression of the variants.

Of note, we also found that clinically applied anti–PD-L1 antibodies 28-8 and 22C3 sometimes showed the faint staining pattern different from E1J2J (Supplemental Figure 4) such as in case #68, which expressed PD-L1–vInt4. This might be due to posttranslational modification like glycosylation or shorter variant expression. Thus, the choice of PD-L1 antibody might affect the evaluation of PD-L1 levels that may impact the therapeutic strategy.

### Expression of PD-L1–vInt4 in other cancer types from the TCGA data set.

**Figure 3:**
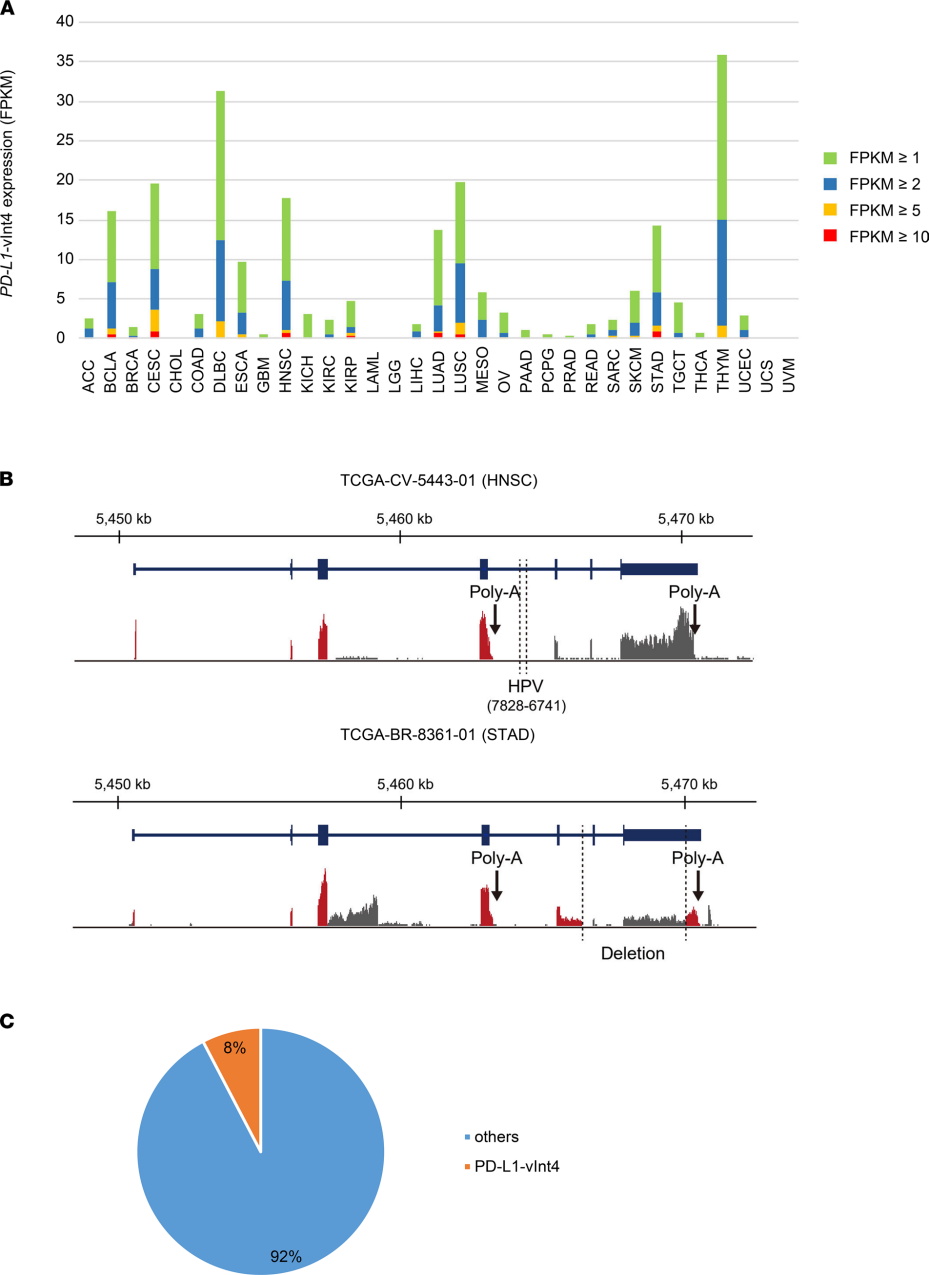
PD-L1–vInt4 expression was found in various types of cancer. (**A**) The percentage of cases expressing PD-L1–vInt4–specific sequence in intron 4 with FPKM ≥ 1, ≥ 2, ≥ 5, and ≥ 10 in each TCGA cancer type. (**B**) Structural changes associated with PD-L1–vInt4 expression in a case of HNSCC (TCGA-CV-5443-01) and STAD (TCGA-BR-8361-01). Aberrant transcripts are colored in red. (**C**) PD-L1–vInt4 was detected in some of the clinical samples. Data were obtained from CCLE.

To investigate whether PD-L1–vInt4 is expressed in other cancer types, we searched the TCGA for the expression of PD-L1–vInt4–specific regions in intron 4 in 10,473 cancer samples from 33 tumor panels for which RNA-Seq data were available. Among them, patients with DLBCL and thymoma showed the highest frequency of PD-L1–vInt4 expression, accounting for one-third of the patients (Figure 3A). In addition, whereas no patients (or only a small proportion) harbored PD-L1–vInt4 expression in approximately two-thirds of all cancer types, as much as 10%–20% of patients with squamous cell carcinoma, including LUSC, head and neck squamous cell carcinoma (HNSCC), and cervical squamous cell carcinoma, expressed PD-L1–vInt4 (Figure 3A). These results suggest that PD-L1–vInt4 expression is prevalent across a variety of cancer types. Of note, a structural change was associated with high expression of PD-L1–vInt4 in 2 patients. In a case of HNSCC (TCGA-CV-5443-01), whole-genome sequencing revealed that human papillomavirus was integrated into intron 4 of PD-L1 (Figure 3B). RNA-Seq also identified a deletion involved in exons 6 and 7 of PD-L1 transcript in a case of stomach adenocarcinoma (STAD; TCGA-BR-8361-01) (Figure 3B). These observations suggest that clonal selection of a structural alteration causes the expression of PD-L1–vInt4 in these cancers.

We also searched for Cancer Cell Line Encyclopedia (CCLE) data sets to investigate whether PD-L1–vInt4 is expressed among tumor cell lines. There were 17 of 222 cell lines that expressed PD-L1–vInt4 (Figure 3C), implying that this variant was not a rarely observed phenomenon.

### PD-L1–vInt4 was stably secreted from the cells.

To evaluate the function of PD-L1–vInt4 in vitro and in vivo, we generated a virus vector that has a conjugated structure of mouse PD-L1 and intron 4 region of human PD-L1, since the mouse intron 4 amino acid sequence before the stop codon is very short, compared with the human intron 4. Furthermore, the mouse intron 4 does not contain cysteine residue that can dimerize through disulfide bond formation, which is the case in the human PD-L1 intron 4–derived sequence in which dimerization is presumed to have a role in its immunosuppressive function (29). For in vitro assessments designed for human cell lines, we used human PD-L1–vInt4, consisting of human PD-L1 exons 1–4 plus human intron 4. MC38 and PC9 cells were infected with the lentivirus encoding murine PD-L1 plus human PD-L1–vInt4 (mh), murine PD-L1 plus murine PD-L1–vInt4 (mm), and human PD-L1 plus human PD-L1–vInt4 (hh). It was confirmed that they stably expressed PD-L1–vInt4 in the mRNA (Supplemental Figure 5) and protein (Supplemental Figure 6) levels. Both human and murine PD-L1–vInt4 variants were stably secreted in cell culture media. In addition, PD-L1 protein expression in mouse tumors was visually confirmed by immunofluorescence staining, after treating with brefeldin A to temporarily inhibit PD-L1–vInt4 secretion by stopping membrane trafficking (Supplemental Figure 7). We could not compare the full-length PD-L1 to the secreted type of PD-L1 because we did not have an intracellular domain–recognizing anti–PD-L1 antibody for mice.

### PD-L1–vInt4 does not demonstrate an immunosuppressive function in vivo but works as a decoy for the anti–PD-L1 antibody.

**Figure 4:**
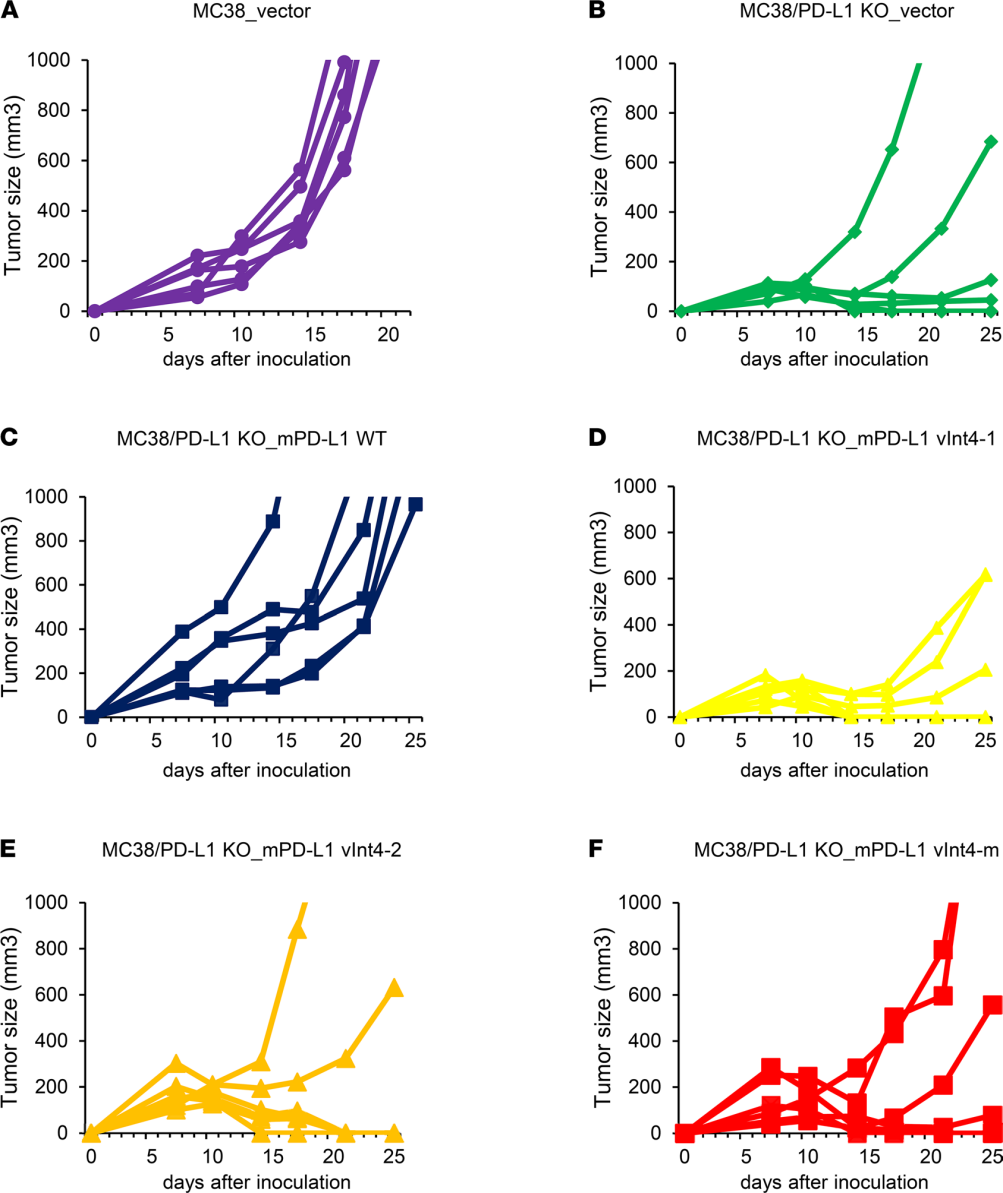
Overexpression of soluble PD-L1 variants did not help the tumor growth alone. (**A**) Mice were injected with 1 × 10^6^ MC38 cells transduced with empty vector (*n* = 6). (**B**) MC38_PD-L1–KO cells transduced with empty vector (*n* = 6). (**C**) MC38_PD-L1–KO cells transduced with murine WT PD-L1 (*n* = 6). (**D**) MC38_PD-L1–KO cells transduced with mh #1 (*n* = 6). (**E**) MC38_PD-L1–KO cells transduced with mh #2 (*n* = 6). (**F**) MC38_PD-L1–KO cells transduced with mm (*n* = 6). Each line represents the tumor volumes for each mouse. mh #1 and #2 are different clones. Each group was compared with 2-sided Mann-Whitney *U* test.

To investigate whether splice variant PD-L1–vInt4 demonstrates any immunosuppressive function on its own, we overexpressed PD-L1 WT/mh/mm on PD-L1–KO MC38 cells (MC38_PD-L1–KO). Each overexpressed cell was injected into the flanks of mice, and tumor growth rates were compared. It is broadly known that PD-L1 plays a positive role in tumor growth (30), and we confirmed significant interference in tumor growth when PD-L1 was knocked out from MC38 cells, which implies that PD-L1 works as an immune checkpoint, helping the MC38 tumor to escape from immune surveillance (Figure 4, A and B). Tumor growth recovered when PD-L1 was overexpressed in MC38_PD-L1–KO cells (Figure 4C), thus validating that the growth suppression seen in MC38_PD-L1–KO tumors was dependent on PD-L1, which is in concordance with other reports (30). When evaluated among variant-overexpressed tumors, secreted PD-L1 alone did not produce any significant differences in tumor growth rates, compared with the vector (Figures 4, D–F). These results suggest that possessing a transmembrane domain and cell surface–expressed WT PD-L1 is required for escaping attack by immune cells and that soluble PD-L1 variant expression alone does not exert an immunosuppressive function.

Next, we performed PD-1/PD-L1 blockade bioassay to investigate the activity of PD-L1–vInt4 in vitro. In this bioassay system, TCR signaling can be monitored by NFAT-luciferase–mediated (NFAT-luc–mediated)luminescence in Jurkat cells, mimicking effector T cells; the inhibition of NFAT-luc activity by PD-1/PD-L1 interaction can be monitored by the overexpression of *PDCD1* in Jurkat cells and *PD-L1* overexpression in artificial APC/CHO-K1 (aAPC/CHO-K1) cells (K1), mimicking antigen-presenting cells. When this PD-1/PD-L1 interaction is blocked with an anti–PD-L1 (αPD-L1) or an αPD-1 antibody, increased luminescence should be detected.

**Figure 5:**
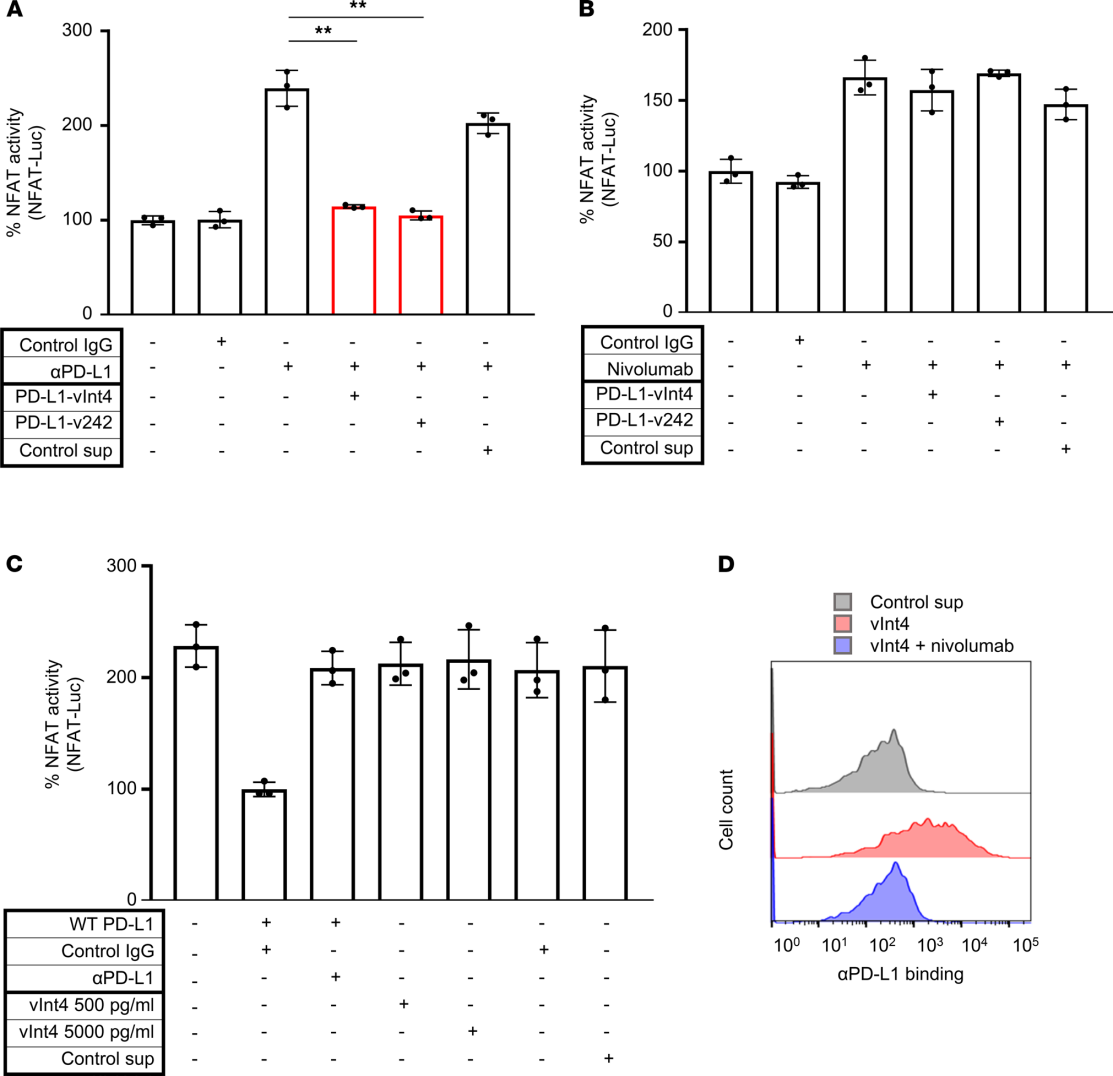
PD-L1–vInt4 demonstrated its function as a decoy in vitro. (**A**) Evaluation of NFAT activity by measuring luminescence when PD-1 effector cells preincubated with αPD-L1 and secreting PD-L1 variants were cocultured with K1_PD-L1 WT cells. PD-L1–v242 and αPD-L1 were added in 3:1 molar ratio, whereas the ratio was 6:1 with PD-L1–vInt4 and αPD-L1. *n* = 3. **P < 0.01 by paired 2-tail Student’s *t* test. (**B**) Comparison of NFAT activity by measuring luminescence — this time using PD-1 effector cells preincubated with nivolumab and secreting PD-L1 variants. Secreted variants do not significantly suppress the signaling; paired 2-tail Student’s *t* test. Each condition was compared with the third bar counted from the left end in **A** and **B**. (**C**) Evaluation of NFAT activity by measuring luminescence. PD-1 effector cells were cocultured with K1 or K1_PD-L1 WT cells. PD-L1–vInt4 was added at a physiologically plausible concentration and at a 10-fold concentration. Secreted variants do not significantly suppress the signaling; paired 2-tail Student’s *t* test. Each condition was compared with the bar in the left end in **C**. Data represent mean ± SEM in **A**–**C**. (**D**) Jurkat–PD-1 cells incubated with or without Fc-tagged PD-L1–vInt4 were evaluated with FACS. As for nivolumab, it was added 30 minutes before PD-L1–vInt4.

We first confirmed that this system worked as expected, by observing that *PDCD1*-overexpressed Jurkat cells (Jurkat–PD-1) showed increased luminescence, implying credible interaction with *PD-L1*–overexpressed aAPC/CHO-K1 cells (K1_PD-L1) (Figure 5A). No change in NFAT-luc activity was observed when PD-L1–vInt4 (collected from the supernatant of PD-L1–vInt4–overexpressed PC9 cells) was added to K1 cells. This implied that PD-L1–vInt4 alone did not have an immunosuppressive function. We then added PD-L1–vInt4 with αPD-L1 to K1_PD-L1 cells, and luminescence was suppressed to the same level as in the control IgG when the amount of PD-L1–vInt4 was twice as great as that of αPD-L1 (Figure 5A). Of note, PD-L1–v242 suppressed luminescence with the equivalent amount of substance to αPD-L1, which implied that PD-L1–vInt4 needed to dimerize for trapping αPD-L1 (Figure 5A). After adding nivolumab, a major αPD-1 in clinical use, we did not observe any significant suppression of luminescence, which implied that PD-L1–vInt4 worked as a decoy against αPD-L1 (Figure 5B). We also observed that the mere binding of PD-L1–vInt4 to Jurkat–PD-1 cells did not suppress the TCR signaling, and the binding was inhibited by nivolumab (Figure 5, C and D).

### vInt4 exhibits resistance against αPD-L1 treatment in vivo.

**Figure 6:**
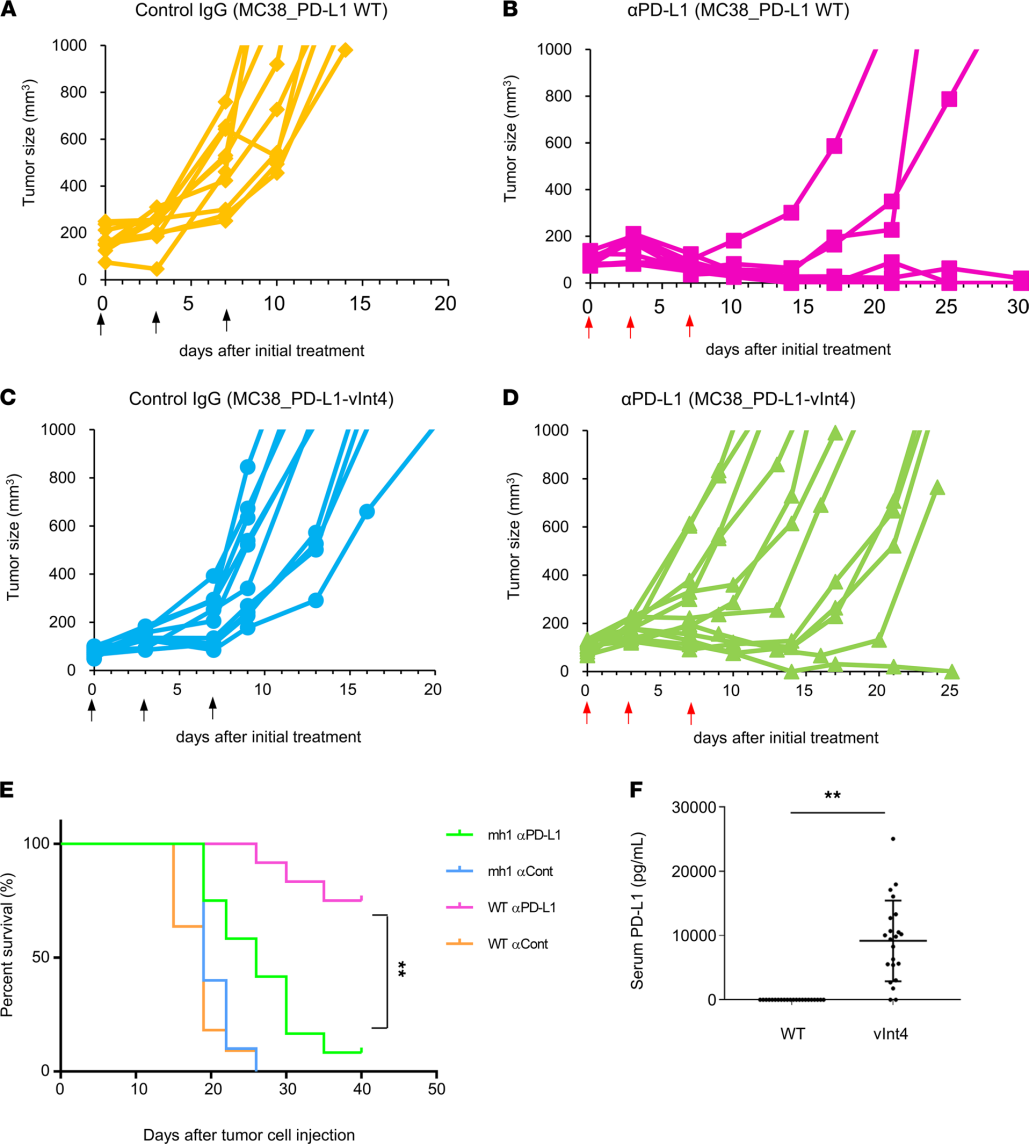
PD-L1–vInt4-overexpressed mice displayed resistance in αPD-L1 treatment. (**A**–**D**) Mice were injected with 5 × 10^5^ MC38 cells with overexpression of WT PD-L1 or PD-L1–vInt4 and treated with 75 μg of anti–PD-L1 or control IgG 3 times a week by i.p. injection. *n* = 10–12 mice/group. Similar experiments were performed 3 times. Each line represents the tumor volumes for each mouse. Each figure represents a mice group of: (**A**) MC38_mPD-L1 WT, treated with control IgG; (**B**) MC38_mPD-L1 WT, treated with αPD-L1; (**C**) MC38_mPD-L1–vInt4, treated with control IgG; or (**D**) MC38_mPD-L1–vInt4, treated with αPD-L1. The regimen of treatment is indicated by black arrows for control IgG and red arrows for αPD-L1. (**E**) Kaplan-Meier curves of treated mouse groups in **A**–**D**. **P < 0.01 by Gehan-Breslow-Wilcoxon test. (**F**) Serum PD-L1 levels of mice in experiments **A**–**D** were sequentially measured by ELISA. Sera were collected just before the endpoint of the experiment or at sacrificing. Data represent mean ± SEM. **P < 0.01 by 2-sided Mann-Whitney *U* test. Similar experiments are conducted twice for **A**–**F**.

To investigate whether PD-L1–vInt4 also acts as an immunosuppressant in vivo, we inoculated mh– or WT–PD-L1–overexpressed MC38 cells s.c. into the flanks of mice and developed their tumors to compare their growth rates under αPD-L1 treatment. MC38_PD-L1–vInt4 (mh) tumors significantly outgrew MC38_WT tumors, despite αPD-L1 treatment. The expression of PD-L1–vInt4 was significantly lethal when mice were treated with αPD-L1 (Figure 6, A–D); WT PD-L1–overexpressed mice responded to αPD-L1 treatment, and the survival rate was significantly better than that of PD-L1–vInt4–overexpressed mice (Figure 6E). This phenomenon corresponded to the result we obtained in vitro and implied that PD-L1–vInt4 also works as a decoy in vivo. The reason why some tumors shrank slightly at first and were delayed in growth, or responded completely, can be explained by the accumulation of internal PD-L1–vInt4 secreted by the tumors. Tumor proliferation was suppressed when the amount of αPD-L1 was increased (Supplemental Figure 8). However, they were not sufficient to exterminate the tumor cells permanently, probably because of their continuous secretion of PD-L1–vInt4 finally overwhelmed the αPD-L1 and resulted in regrowth of the once-shrunk tumors. The level of PD-L1–vInt4 is believed to increase over time (Figure 6F); thus, antibodies become invalid when they are numerically overwhelmed and completely decoyed. Although the same effect was observed with PD-L1–v242, which had worked as a decoy in monomer form, in vitro examination implied that PD-L1–vInt4 might act as a decoy in dimer form. Therefore, tumor cells would need to secrete double the amount of PD-L1–v242. As a result, PD-L1–vInt4 was observed to be a relatively weaker decoy.

## Discussion

Tumor resistance to ICIs is a major problem, with low response rates due to tumors acquiring certain mechanisms of immune tolerance. Certain specific side effects may be related to immune activation. Increase and augmentation of side effects can occur when ICIs were used in combination therapy with conventional anticancer agents.

In this study, we first demonstrated that we were able to detect the splice variants (possibly the secreting ones) in LUSC samples by evaluating IHC. We used 2 different antibodies that detect intracellular and extracellular domains — a method that can easily be applied in clinical assessment. Several different antibodies are used clinically to evaluate PD-L1 levels, such as 22C3, used for measuring the tumor proportion score (TPS) before starting treatment with pembrolizumab. However, their accurate recognition sites and the existence of soluble variants are not usually considered, although significant differences in the positive rates have been highlighted by some studies (31–33). It has been reported that intracellular domain–recognizing antibody SP142 shows lower PD-L1 levels in IHC (34), implying the prevalent existence of secreted splicing variants among tumors, which might be explained by differences in the recognizing domains. Once some splicing variants are detected, discussion on their function should naturally follow.

We next confirmed that PD-L1–vInt4 worked as decoy molecule in secreted PD-L1, aside from PD-L1–v242. We demonstrated that secreted variants such as PD-L1–vInt4 are able to decoy αPD-L1, virtually attenuating the effect of αPD-L1. Although it is previously reported that PD-L1–vInt4 can act suppressively to T cells (29), the secretion alone did not affect the tumor growth in vivo (Figure 4, D–F), and PD-L1–vInt4 binding to TCRs did not seem to stimulate the receptors or the pathway below and as we saw in Figure 5C. We hypothesized that the presence or absence of the formation of microclusters in TME (23), probably because of PD-L1–vInt4 lacking transmembrane region, make the results appear different; PD-L1–vInt4 molecules apparently reduce T cell proliferation or IFN-γ production in vitro, but they cannot work effectively against T cells in vivo because they do not form microclusters to affect TCR signaling. It is also reported that PD-L1 on DCs suppresses T cell activity and T cells restore their activity when PD-L1 on DCs are inhibited (35). Another study reports that PD-L1 on host antigen presenting cells is essential when responding to PD-L1 blockade (36). These may also be explained by the results that showed that PD-L1–vInt4 did not stimulate TCR signaling just by binding to them (Figure 5, C and D). It is clinically suggestive and meaningful that it is possible to overcome this type of resistance simply by increasing the amount of αPD-L1. However, simply increasing the dose of αPD-L1 was not sufficient to bring the animals into complete response in our in vivo model (Supplemental Figure 8), probably because the produced PD-L1–vInt4 overwhelmed the amount of αPD-L1, along with the proliferation of tumor cells. Supplemental Figure 8 shows that simply increasing the dose of αPD-L1 is at least partially effective as a further antitumor immunotherapy. Combination with other antitumoral drugs or switching into αPD-1 treatment may be the appropriate strategies in the future. We also observed that staining patterns differ when secreted PD-L1 splice variants exists, implying that clinicians can take the IHC staining pattern into account when adjusting the dose of αPD-L1, which is preferable in terms of cost-effectiveness in some cases. Examining serum PD-L1 concentration can also help clinicians to judge whether soluble variants exist. We proved this to be possible in a murine model, as shown in Figure 6F and Supplemental Figure 9. A measurement method for serum soluble PD-L1 level in humans is already available; it is reported that serum PD-L1 level correlates with patient prognosis (37–40) and clinical response (41). We also showed the concentration level of secreted PD-L1 in clinical samples such as serum and pleural effusion in our previous study (28), and its level seemed to be mutually compatible with our murine samples if they are collected at some time point when the tumors are within plausible sizes when assumed in real patients (Supplemental Figure 9).

Despite that PD-L1 expression is considered a predictive biomarker for αPD-1/αPD-L1 treatment (42), cases with discordance between the pretreatment PD-L1 IHC score and treatment responsiveness are frequently seen in clinical situations, which is a major problem (43, 44). PD-L1 expression is not necessarily a sufficiently reliable or credible index to judge responsiveness, even though its evaluation is usually required before treatment in several regimens. The decoy effects of PD-L1–v242 and PD-L1–vInt4 might provide a possible explanation for the fact that PD-L1 TPS in IHC does not necessarily predict the responsiveness to αPD-L1 treatment (45), as only extracellular domain–recognizing antibodies are used to determine the TPS. It has recently been reported that PD-L1 expression levels differ between primary tumors and paired distant metastases (46, 47). A possible reason is that their expression levels in soluble splice variants may change depending on the location of the metastases, the change in microenvironment, and the timing of dissemination. We also looked into the postoperative courses of the patients in Table 1 to investigate whether serum level of PD-L1 and prognosis correlate. Fortunately, most of the patients are surviving without recurrence, and they have not received further treatment. We went through for other cases and found several cases started their treatment with atezolizumab. In cases assessed as progressive disease after several courses of atezolizumab treatment, we revealed that PD-L1 staining by SP142 fell off in their protreatment specimens, while E1J2J staining did not (Supplemental Figure 10A), which means the existence of soluble PD-L1 before treatment, whereas a case with partial response did not show a significant difference between E1J2J and SP142 (Supplemental Figure 10B). PD-L1 proteins are known to harbor several N-glycosylation sites, which are known to have a role on their stabilization (48). However, their half-lives are not compared when the tumor specimens are fixed into FFPE blocks; the difference in half-lives can affect the evaluation score because not all of the specimens are stained or scored at the same time and the degree of their loss of expression may differ (49, 50).

Mainstream anticancer therapies, including current regimens for lung cancers, combine ICIs with platinum antitumor agents or other cytotoxic drugs. It would be helpful for clinicians if some cases could be dealt with simply by regulating the doses of the drugs. Further studies are needed to assess whether simply increasing the amount of αPD-L1 can improve some indices of patient prognosis. Some studies have reported concordance among several αPD-L1s (51), whereas others have reported inconsistencies (31, 52, 53). Soluble variants might be one reason for these different conclusions.

**Figure 7:**
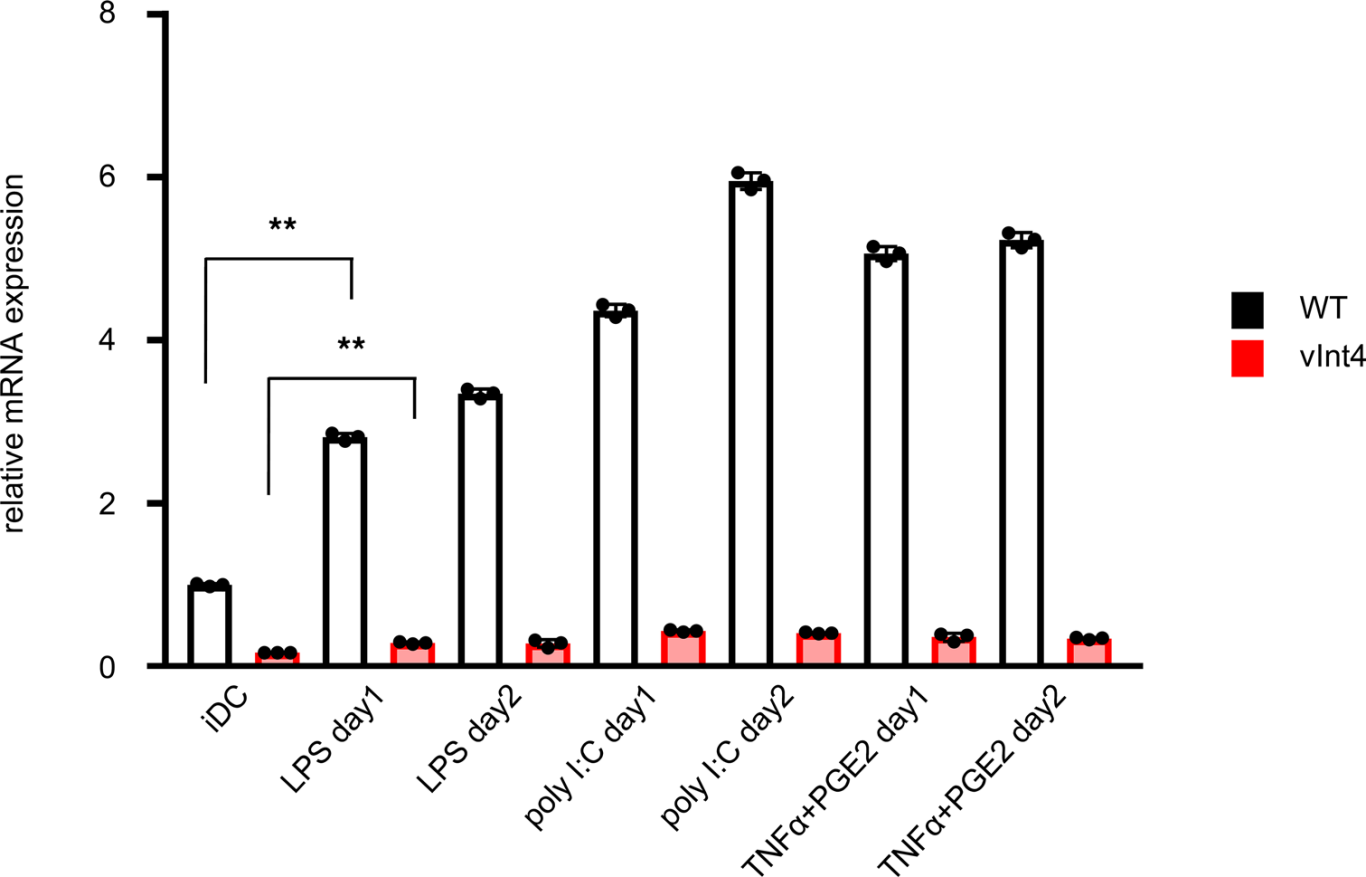
PD-L1–vInt4 was induced in normal human DCs. The mRNA levels were evaluated in normal human DCs. LPS, poly I:C, or TNF-α + PGE2 were added and incubated for 24 or 48 hours. iDC, immature DCs. **P < 0.01 by paired 2-tail Student’s *t* test. Each condition is compared with the expression level in iDC, which is shown by the bar in the left end; WT and vInt4 are assessed independently. Data represent mean ± SEM.

We must remember that it is reported that some nonmalignant cells, such as DCs, also produce soluble PD-L1 (54). When DCs were treated with PD-L1–inducing cytokines or chemicals, PD-L1–vInt4 mRNA were actually induced simultaneously with WT PD-L1 (Figure 7). Taking into account the fact that DCs are one of a relatively scarce populations in serum or TME, the amount and its effect of soluble PD-L1 secreted by DCs might be limited when compared with tumor cells.

This study has some limitations. We need to investigate the correlation between the results of RNA-Seq and of IHC to determine the existence of splice variants and whether immunosuppressive effects are observable in splice variants other than PD-L1–v242 and PD-L1–vInt4. It is not known whether other splice variants, such as PD-L1–v229 and PD-L1–v178, may play any role in constructing an immunosuppressive environment. Our IHC analysis is limited owing to the unavailability of a complete list showing recognition sites for each antibody, making it difficult to differentiate each splice variant. Therefore, we cannot obtain detailed information beyond the existence of other secreted splice variants. Heterogeneity of the tumor itself is also a problem in some cases, as samples are obtained and evaluated only partially (55). It seems that PD-L1–v242 and PD-L1–vInt4 work as decoys for αPD-L1, though little is known about other variants; their function should also be assessed in the future. Dimerization is a unique, characteristic mechanism that explains the decoy effect of the variants in αPD-L1 treatment as a decoy. However, there might be other molecular structural mechanisms for achieving immunosuppression.

We did not assess interaction with immune-related cells, such as T cells or macrophages. Further investigation is needed as to whether PD-L1 splice variants have a direct impact on these immune cells.

In short, we have demonstrated that IHC can be a tool for predicting the existence of soluble variants and that their existence might provide a compelling explanation for the differing staining pattern among several antibodies and for the discrepancy between the IHC score and immunotherapy responsiveness. PD-L1–vInt4 works as a decoy for αPD-L1, when dimerized, and might affect treatment strategies, such as the doses of PD-L1.

## Methods

### IHC staining.

IHC staining was performed on representative surgical tissue sections from FFPE tissue blocks of LUSC patients using anti–human PD-L1 antibodies: E1J2J, Cell Signaling Technology; 28-8, Abcam; 22C3, Dako; SP142, Spring Bioscience; anti–human CD8 antibodies (C8/144B; Nichirei Bioscience); anti–human PD-1 antibodies (NAT105; Abcam); anti–human β2M antibodies (A0072; Dako); and anti–human HLA class I-A/B/C antibodies (EMR8-5; Hokudo). The detection of immunostaining was performed using Histofine Simple Stain MAX-PO (Nichirei Bioscience) or BOND polymer Refine Detection kit (Leica Biosystems). Some of the sections were stained with the PD-L1 22C3 pharmDx kit (Dako). PD-L1 positivity was defined as membranous or cytoplasmic staining in at least 1% of tumor cells. The staining results of β2M and HLA class I-A/B/C of tumor cells were categorized as negative (0%), focally positive (<50%), or positive (≥50%).

### Immunofluorescence staining.

Immunofluorescence staining was performed on formaldehyde-fixed cells. After permeabilization with Blocking One Solution (Nacalai Tesque) containing 0.3% Triton X-100 for 1 hour, the cells were labeled with αPD-L1 antibodies E1J2J (15165, Cell Signaling Technology), 28-8 (ab205921m, Cell Signaling Technology), SP142 (M4424, Spring Bioscience), and anti–mouse PD-L1 (AF1019, Abcam) overnight at 4°C. As secondary antibodies, Alexa Fluor 488 (A11034; Thermo Fisher Scientific) and Alexa Fluor 594 (A11080; Thermo Fisher Scientific) were applied after washing the primary antibodies off with PBS. Nuclei were stained with Hoechst 33342 (Thermo Fisher Scientific). Images were captured using a FLUOVIEW FV1000 laser scanning microscope (Olympus) at 60× magnification.

### Evaluating transcripts of PD-L1 variants.

Raw reads were preprocessed by removing Illumina adapter sequences and low-quality bases using Trimmomatic-0.39 software (56) with the following options: LEADING, 10; TRAILING, 10; SLIDINGWINDOW, 4:20; MINLEN, 40. The quality-controlled reads were aligned onto the Human genome sequence (UCSC hg38) by HISAT2 (57), and the SAM file obtained was converted to a BAM file using SAMtools v1.9 (58). To estimate expressions of all possible PD-L1 transcripts, all reads aligned at the *PD-L1* genomic region chr9:5456103–5467947 were extracted from the BAM file using SAMtools and were assembled using StringTie v2 (59), without reference annotation. Then, expressions of all *PD-L1* transcripts were estimated by using the assembled transcript data in StringTie with the “-e” option. Estimated expressions were compared using Ballgown (60).

For CCLE data set analysis, we obtained data from GDC legacy portal (https://portal.gdc.cancer.gov/legacy-archive/search/f?filters=%7B%22op%22:%22and%22,%22content%22:%5B%7B%22op%22:%22in%22,%22content%22:%7B%22field%22:%22cases.project.program.name%22,%22value%22:%5B%22CCLE%22%5D%7D%7D%5D%7D). The BAM files were estimated in the same way as described above.

### Analysis of TCGA data sets.

We analyzed 10,473 TCGA samples from 33 cancer types, for which RNA-Seq data were publicly available, as previously reported (27). Briefly, RNA-Seq data of these samples were obtained from the Cancer Genomic Hub (https://gdc.cancer.gov/) and analyzed using the Genomon pipeline (https://genomon.readthedocs.io/ja/latest/). To search for *PD-L1*–vInt4 expression, we calculated expression of the PD-L1–vInt4–specific region in intron 4 (chr9: 5,463,121–5,463,221). Structural changes and cancer-related viral integration within or near the *PD-L1* gene were investigated, as previously described (27).

### Cell lines.

PC9, Jurkat, and CHO cells obtained from ATCC were cultured in RPMI-1640 with 10% FBS, 100 U/mL penicillin, and 100 μg/mL streptomycin. MC38 cells obtained from Kerafast were cultured in DMEM (low glucose) with FBS, 2 mM glutamine, 0.1 mM nonessential amino acids, 1 mM sodium pyruvate, 50 μg/mL gentamycin sulfate, 100 U/mL penicillin, and 100 μg/mL streptomycin. Normal human DCs obtained from Lonza Pharma & Biotech (CC-2701) were cultured in LGM-3 medium (CC-3211; Lonza Pharma & Biotech), 800 U/mL GM-CSF (215-GM-010; R&D Systems), and 800 U/mL IL-4 (204-IL-010; R&D Systems). To stimulate DCs, LPS (L3024; Sigma-Aldrich), poly I:C (P1530; Sigma-Aldrich), TNF-α (210-TA-020; R&D Systems), and PGE2 (P0409; Sigma-Aldrich) were added as indicated in the previous report (54). Clinical cell lines were established from the tumor samples described below.

### Tumor samples.

Clinical LUSC samples were obtained from patients who underwent pulmonary tumor resection at the Cancer Institute Hospital of the JFCR. All patients provided informed consent for genetic and cell biological analyses. Analyses were performed in accordance with protocols approved by the IRB of JFCR.

### Establishment of PD-L1–KO cells and PD-L1 variant–overexpressing cells.

The CRISPR/Cas9 method (61) was used for the KO of PD-L1. Referring to a previous report (62), we designed a gRNA sequence for exon 1 of the murine *CD274* gene encoding PD-L1 protein (gRNA: 5′-GCTTGCGTTAGTGGTGTACT-3′, 5′-GTATGGCAGCAACGTCACGA-3′). This was cloned into a gRNA cloning vector (62988; Addgene). Then, MC38 cells were transfected individually with these plasmids and Cas9 WT (41815; Addgene), using the Lipofectamine 3000 protocol (Thermo Fisher Scientific). After transfection, cells were sorted by FACS twice to pick up the PD-L1^–^ population.

The same PD-L1 WT– and PD-L1–v242–overexpressed PC9 and MC38 cells were used as in our previous report (28). We established human/murine vInt4-overexpressing cells in this study. To generate pENTR-mPD-L1–vInt4-m, pENTR-mPD-L1–vInt4-h, and pENTR-hPD-L1–vInt4-h, site-directed mutagenesis was performed using the following primers: forward 5′-GAGCTGATTCATCCCAGGTGAGTTGCCTAACTCGTCCCCGGATTCCTAGAAGGGTGGGCGCGCCGAC-3′ and reverse 5′-GTCGGCGCGCCCACCCTTCTAGGAATCCGGGGACGAGTTAGGCAACTCACCTGGGATGATCAGCTC-3′for mPD-L1–vInt4-m, forward 5′-GAGCTGATTCATCCCAGGTAATATTCTGAATGTGTCCATTAAAATATGTCTAACACTGTCCCCTAGCACCTAGAAGGGTGGGCGCGCCGAC-3′ and reverse 5′-GTCGGCGCGCCCACCCTTCTAGGTGCTAGGGGACAGTGTTAGACATATTTTAATGGACACATTCAGAATATTACCTGGGATGATCAGCTC-3′ for mPD-L1–vInt4-h, and forward 5′-CTGAATTGGTCATCCCAGGTAATATTCTGAATGTGTCCATTAAAATATGTCTAACACTGTCCCCTAGCACCTAGAAGGGTGGGCGCGCCGAC-3′ and reverse 5′-GTCGGCGCGCCCACCCTTCTAGGTGCTAGGGGACAGTGTTAGACATATTTTAATGGACACATTCAGAATATTACCTGGGATGACCAATTCAG-3′ for hPD-L1–vInt4-h. pLenti6.3 lentiviral vectors were produced by LR cloning (Thermo Fisher Scientific), and those vectors were then transfected to 293FT cells with Lipofectamine 2000 (Thermo Fisher Scientific). The lentiviruses produced were collected and infected with polybrene (Sigma-Aldrich) to MC38 and PC9 cells. Forty-eight hours after lentivirus infection, cells were selected using blasticidin at the concentration of 5 μg/mL for MC38 and at 10 μg/mL for PC9 cells for 5–7 days. We utilized Fc-tagged PD-L1 plasmid produced in our previous study (28) to generate Fc-tagged PD-L1–vInt4 by site-directed mutagenesis using the following primers: forward 5′-CAGCTGAATTGGTCATCCCAGGTAATATTCTGAATGTGTCCATTAAAATATGTCTAACACTGTCCCCTAGCACCTCGAGCACCATGGTTAGATCTG-3′ and reverse 5′-CAGATCTAACCATGGTGCTCGAGGTGCTAGGGGACAGTGTTAGACATATTTTAATGGACACATTCAGAATATTACCTGGGATGACCAATTCAGCTG-3′.

We performed Sanger sequencing following the manufacturer’s protocol of the BigDye Terminator v3.1 Cycle Sequencing Kit (Thermo Fisher Scientific).

### quantitative PCR analysis.

To quantify the level of mRNA in the cell lines, cDNA, Fast SYBR Green Master Mix (Roche Life Science), and primers (PD-L1: forward 5′-TGGCATTTGCTGACGCATTT-3′ and reverse 5′-TGCAGCCAGGTCTAATTGTTTT-3′; GAPDH: forward 5′-TGCACCACCAACTGCTTAGC-3′ and reverse 5′-GGCATGGACTGTGGTCATGAG-3′; hPD-L1–vInt4-h: forward 5′-ATTAGATCCTGAGGAAAACCATAC-3′ and reverse 5′-GCTAGGGGACAGTGTTAGACATATTT-3′) were mixed. The reaction was performed and recorded by LightCycler 96 system (Roche Life Science). To compare WT PD-L1 and PD-L1–vInt4 expression, we prepared a standard curve using 10-fold serial dilutions of 1:1 mixture of WT PD-L1 and PD-L1–vInt4 plasmids to adjust the difference of the amplification efficiency between the primer sets.

### Purification and concentration of secreted PD-L1.

Secreted PD-L1 was collected from the culture supernatant of PC9 parental cells (as a control supernatant) and PC9-vInt4 cells. The cells were seeded in 15 cm dishes and cultured in an incubator until they reached 80%–90% confluency; then, the media were replaced with serum free RPMI-1640 for 24 hours. When the supernatant was collected, the debris was removed by centrifuging at 2000*g* for 5 minutes at 4°C followed by 10,000*g* for 15 minutes at 4°C. Secreted PD-L1 was concentrated using Vivaspin 20 (Sartorius) with a 10 kDa molecular weight cut-off. Collected concentration was ultracentrifuged at 100,000*g* for 90 minutes at 4°C for removal of particulates, including exosomes.

### Western blotting analysis.

To evaluate the secreted PD-L1 variants by Western blotting, we needed to first collect the secreted variants. We incubated 1 × 10^6^ PC9 or 5 × 10^5^ MC38 cells overexpressing each variant for 24 hours. The culture supernatant was centrifuged at 20,000*g* for 5 minutes at 4°C, followed by 10,000*g* for 15 minutes at 4°C to remove debris and other unnecessary particles, as described in the purification section. Three times the volume of cold acetone was added, and the proteins in the supernatant were precipitated for more than 2 hours at –20°C. The sample tubes were centrifuged at 10,000*g* for 10 minutes at 4°C; then, the precipitation was dried and resuspended in SDS lysis buffer (100 mM Tris, 1% SDS, 10% glycerol, and 10% 2-mercaptoethanol).

Western blotting was performed as previously described (63). The primary antibodies we used were as follows: PD-L1 (Cell Signaling Technology), E1J2J (15165; 1:1000; Cell Signaling Technology), E1L3N (13684; 1:4000; Abcam), 28-8 (ab205921m; 1:2000; Dako), 22C3 (SK006; 1:200; Spring Bioscience), SP142 (M4424; 1:1000; Spring Bioscience); AF1019 (1:400; Abcam), and GAPDH (1:5000; MAB374; MilliporeSigma).

### Flow cytometry analysis.

To test the binding of PD-L1–vInt4 to Jurkat–PD-1 cells, samples were mixed together and preincubated for 2 hours at 4°C. Fc-tagged PD-L1–vInt4 was collected as supernatant of vector-transfected CHO cells incubated for 48–72 hours. Nivolumab was added to Jurkat–PD-1 cells 30 minutes prior to PD-L1–vInt4. Samples were centrifuged at 500*g* for 3 minutes at 4°C and 3 × 10^5^ Jurkat–PD-1 cells were resuspended in 100 μL FACS buffer (PBS with 0.5% BSA). In total, 1 μL of anti–human IgG (goat anti–human IgG–Alexa Fluor 488; A10163; Thermo Fisher Scientific) was added to target Fc-tagged PD-L1–vInt4 and incubated for 30 min at 4°C. After washing with FACS buffer, the cells were resuspended with 500 μL FACS buffer and assayed with FACS Melody (BD Biosciences). The data were analyzed using FlowJo software (TOMY Digital Biology).

### PD-1/PD-L1 blockade bioassay.

The assay was conducted following the manufacturer’s protocol (Promega). Briefly, αPD-L1 or αPD-1 antibodies, as indicated, and PD-1 effector cells (5 × 10^5^ cells/well) were added to K1_PD-L1 cells (4 × 10^5^ cells/well in 96-well plates). To demonstrate that secreted PD-L1 variants decoy antibodies, αPD-L1 or αPD-1 antibody and secreted PD-L1 variants were mixed at the indicated mole ratio for 1 hour at 37°C, followed by coculturing with PD-1 effector cells for 6 hours ahead of addition to K1_PD-L1 cells.

### In vivo mice study.

All mouse studies were conducted according to the protocols approved by the JFCR Committee for the Use and Care of Experimental Animals. A total of 1 × 10^6^ MC38 and PD-L1–KO/overexpressed cells in 100 μL HBSS were s.c. injected into the flanks of 6-week-old C57BL/6 female mice (Charles River Laboratories). Treatment with control IgG (Bio X Cell) and αPD-L1 antibody (10F.9G2; Bio X Cell) was administered i.p. 3 times per week. Tumor volume was calculated using the formula (in mm^3^): (width)^2^ × length/2. The mice were sacrificed when their tumor volume reached 1,000 mm^3^.

### ELISA.

We made use of the DuoSet ELISA kit (R&D Systems) for detection of PD-L1 in mouse serum. First, ELISA plates (96-well; Corning) were coated with capture antibody against PD-L1 overnight at 4°C. Free binding sites were blocked with 200 μL of blocking buffer (1% BSA in PBS) for 2 hours at room temperature. Then, 100 μL of serum sample purified from plasma was added to each well. After washing 3 times with washing buffer (0.05% Tween20 in PBS), 100 μL of streptavidin-HRP dilution was added, followed by incubation for 20 minutes at room temperature. The plates were washed 3 times before adding 1-Step Ultra TMB-ELISA Substrate Solution (100 μL; Thermo Fisher Scientific). Finally, 2N H_2_SO_4_ was added to stop the reaction, and absorbance was measured at a wavelength of 450 nm.

### Statistics.

Paired 2-tailed Student’s *t* tests were applied for the in vitro experiments. Two-sided Mann-Whitney *U* tests were applied to compare 2 groups in in vivo studies. Kaplan-Meier curves were evaluated using the Gehan-Breslow-Wilcoxon test. A 2-tailed value of *P* < 0.05 was considered statistically significant.

### Study approval.

Before surgery, all LUSC patients provided written, informed consent for research use of surgically resected specimens. This study also used surgically resected archive tumor specimens approved by the IRB of the Cancer Institute Hospital of the JFCR. All in vivo studies were conducted according to protocols approved by the Committee for the Use and Care of experimental animals of the JFCR.

## Author contributions

RS, SS, BG, SO, KK, KT, and RK designed the research; RS, SS, BG, AT, and RK performed the in vitro and/or in vivo experiments and analyzed the data. RS, SS, HN, and KT contributed to the IHC analyses. NY, KU, M. Nakao, HN, MM, and M. Nishio collected surgical samples, and they collected and analyzed clinical data. Y. Seto, Y. Shiraishi, and KK performed NGS data analysis. RS, SS, ST, KK, and RK wrote the manuscript. YM, NF, SO, KT, and RK supervised the study.

## Supplementary Material

Supplemental data

## Figures and Tables

**Table 1:**
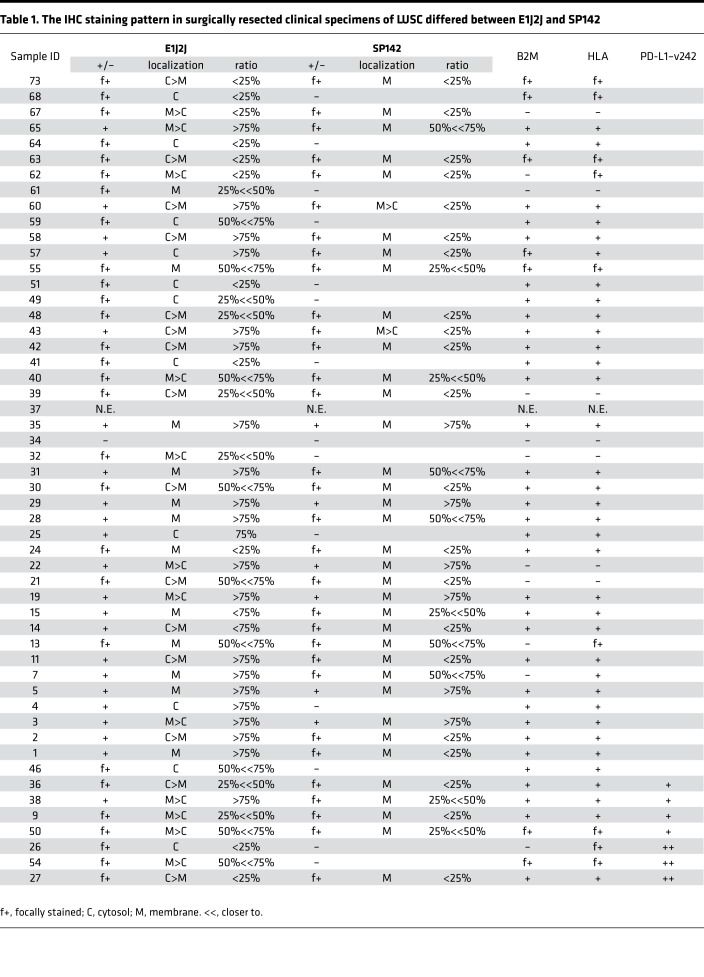
The IHC staining pattern in surgically resected clinical specimens of LUSC differed between E1J2J and SP142

## Version 1 12/07/2021

In-Press Preview

## Version 2 01/11/2022

Electronic publication
