# Supplementary material for: Soluble PD-L1 works as a decoy in lung cancer immunotherapy via alternative polyadenylation
Source: JCI Insight. 2022 Jan 11;7(1):e153323. doi: 10.1172/jci.insight.153323 (PMC8765052; doi:10.1172/jci.insight.153323)
Supplement: Supplemental data [file jciinsight-7-153323-s173.pdf]

Supplementary Information

Soluble PD-L1 through alternative polyadenylation works as a decoy in lung cancer immunotherapy

Ray Sagawa[1,2], Seiji Sakata[3], Bo Gong[1], Yosuke Seto[1], Ai Takemoto[1], Satoshi Takagi[1], Hironori Ninomiya[4], Noriko Yanagitani[5], Naoya Fujita[13], Masayuki Nakao[6], Mingyon Mun[6], Ken Uchibori[5], Makoto Nishio[5], Yasunari Miyazaki[2], Yuichi Shiraishi[7], Seishi Ogawa[8,9,10], Keisuke Kataoka[11,12], Kengo Takeuchi[3,4], Ryohei Katayama[1]*

Supplementary Figure S1

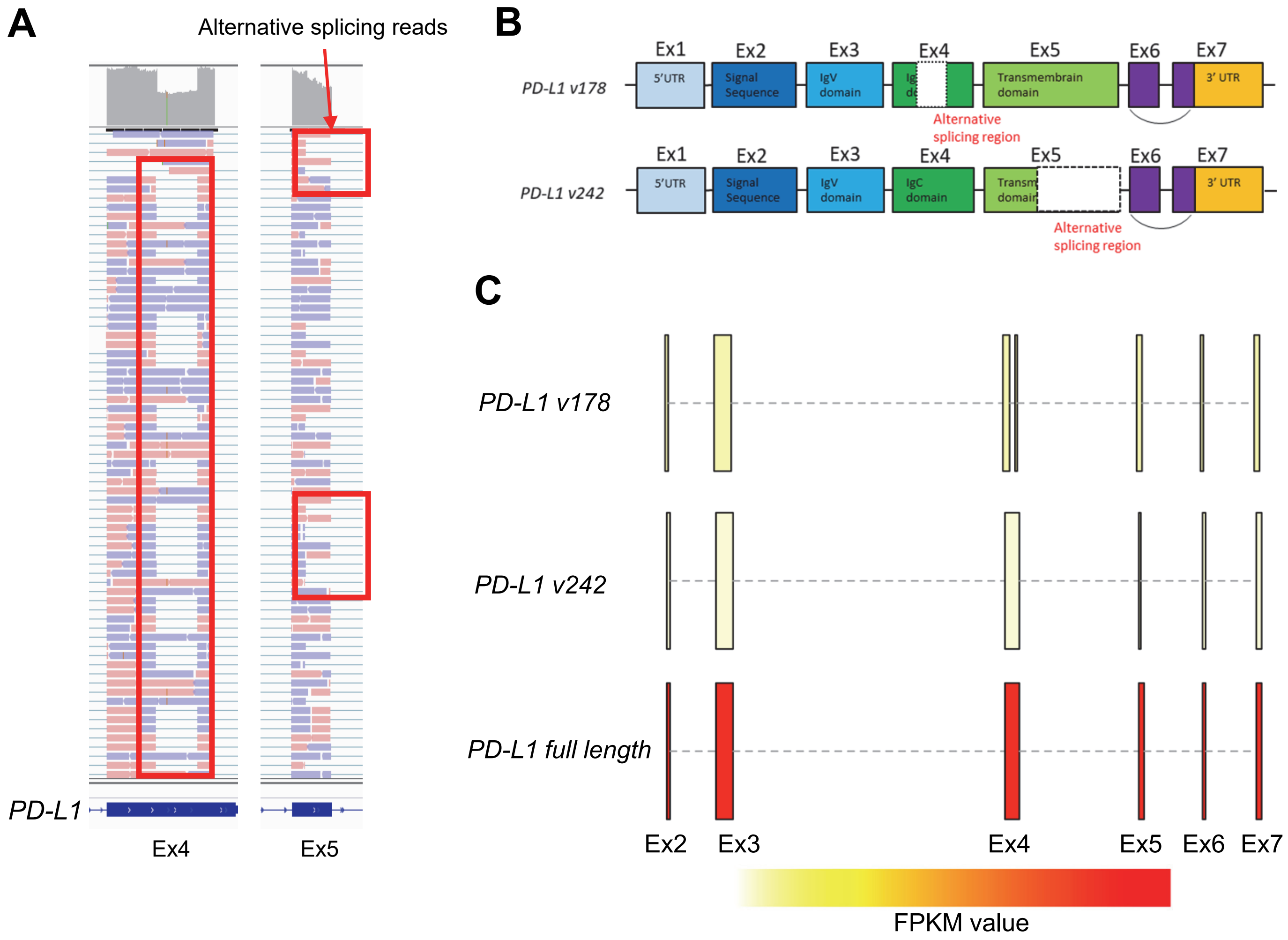

Supplementary Figure S1. Methodology of PCR based PD-L1 variants ultradeep sequencing. (A) IGV view data of the mapped sequence reads on PD-L1 exons 4 and 5. (B, C) Schematic diagram of PD-L1 variants identified by PCR based deep sequencing.

Supplementary Figure S2

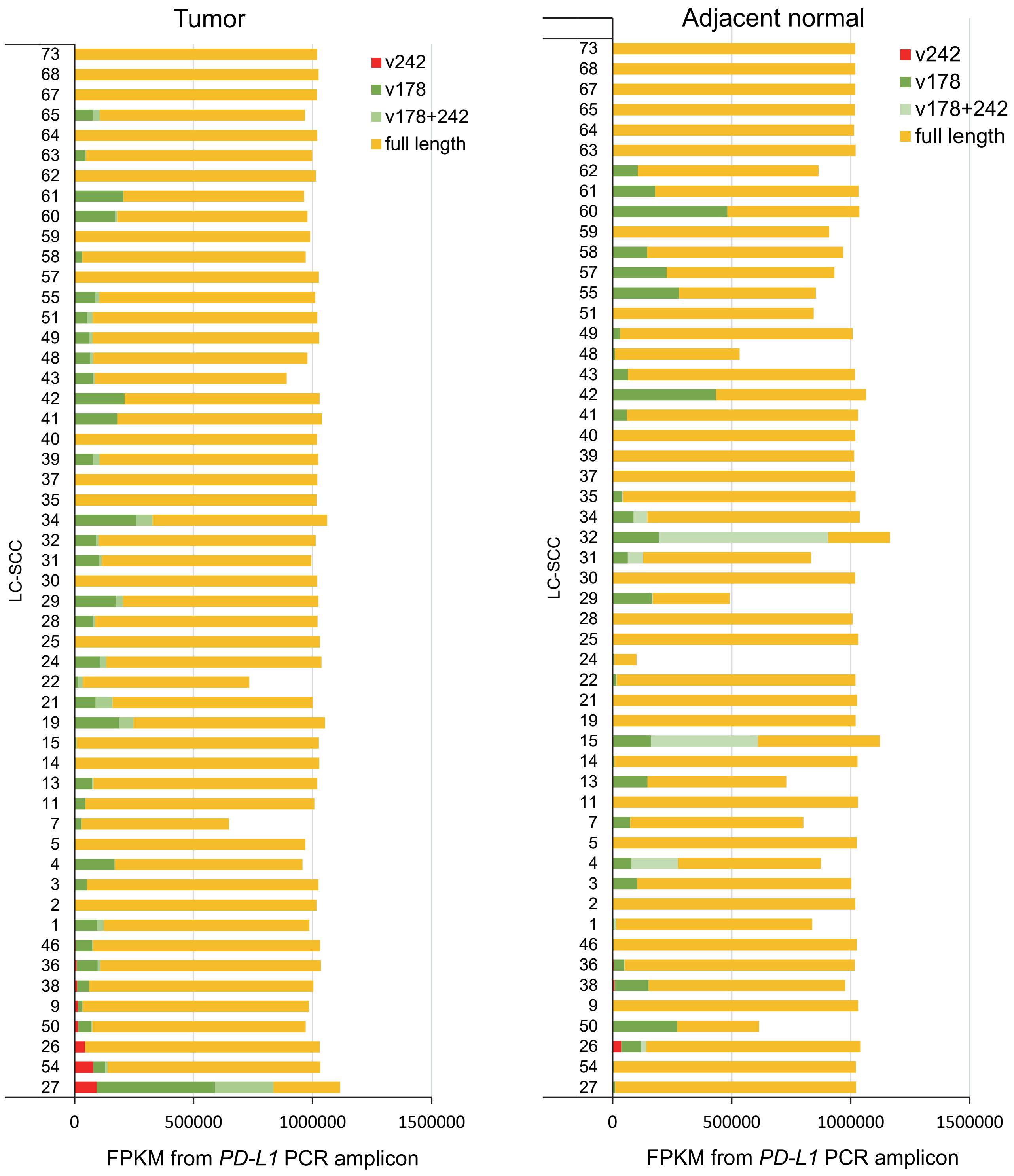

Supplementary Figure S2. Some LUSC cases expressed PD-L1 splice variants. Predicted sequence reads of PD-L1 variants containing exons 2–7 were analyzed by amplicon deep sequencing. This method does not reflect the existence of the PD-L1-vInt4 variant because of the lacking exon 7 and the addition of an alternative poly A at intron 4. Each color bar represents: red, v242; green, v178; light green, v178+v242; yellow, full-length PD-L1.

Supplementary Figure S3

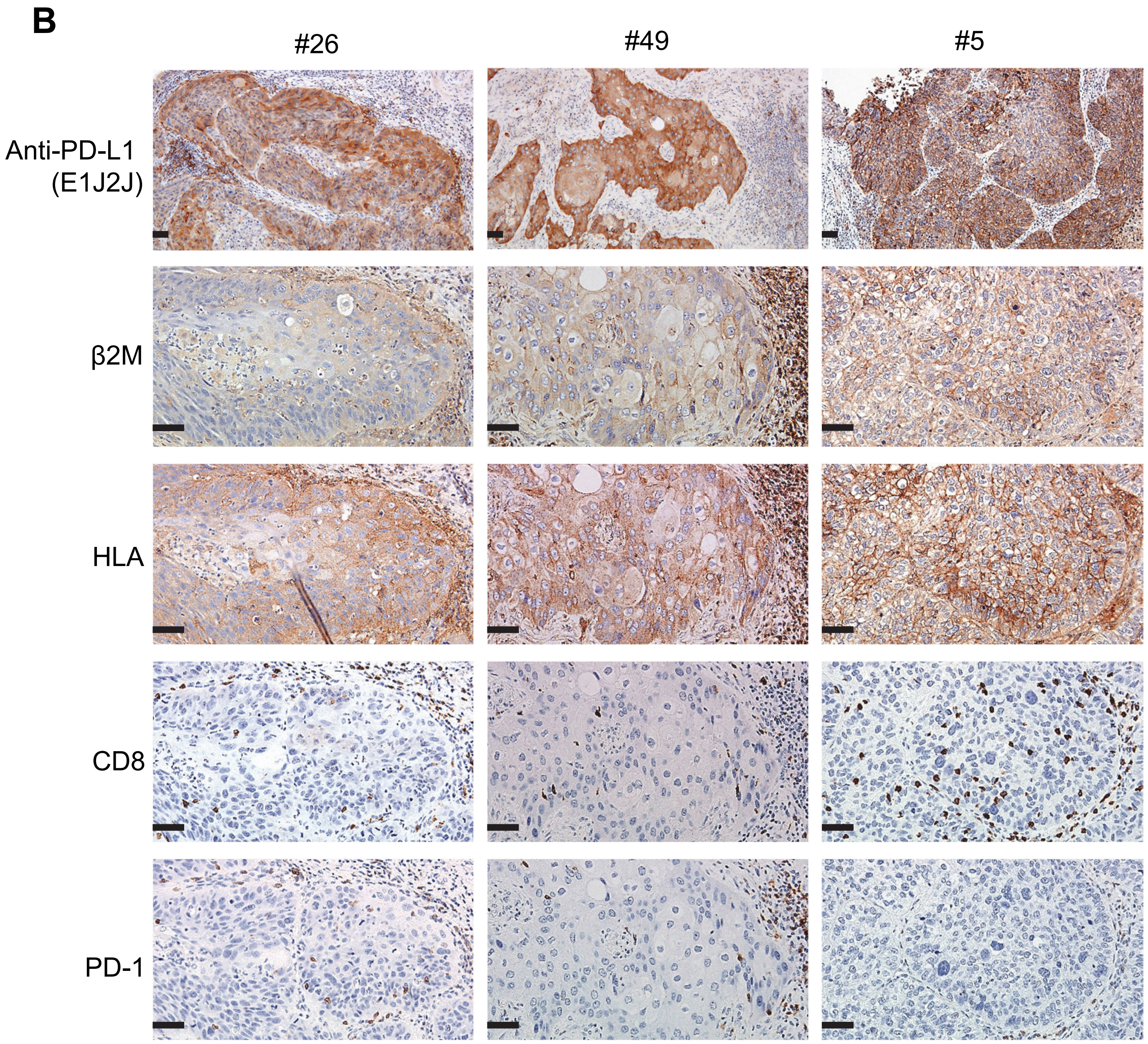

Supplementary Figure S3. **PD-L1 staining variation in different antibodies.** A few PD-L1 positive samples of LUSC were stained with β2M, HLA, CD8, and PD-1. Each scale bar displays a length of 50 μm.

Supplementary Figure S4

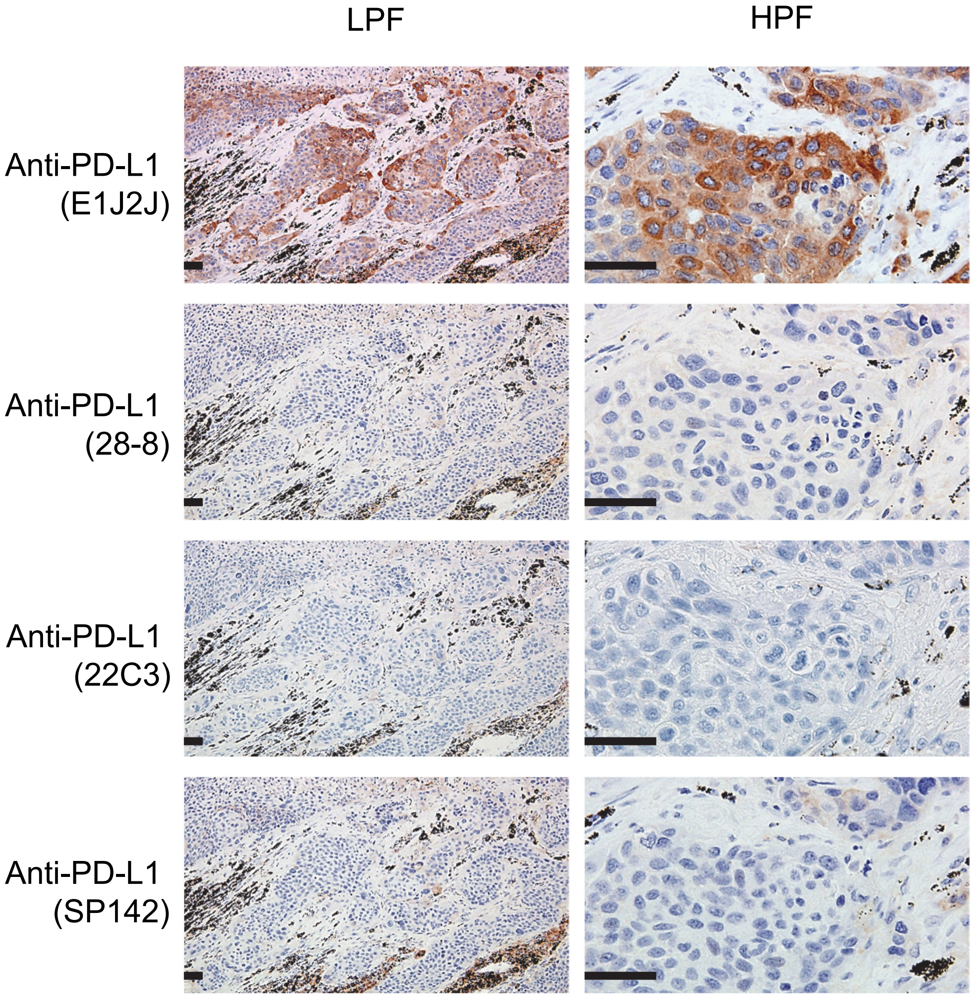

Supplementary Figure S4. **PD-L1 staining varies in different-site-recognizing antibodies.** A clinical sample of LUSC (#68) was stained with several different anti-PD-L1 antibodies. Each scale bar displays a length of 50 μm. LPF: low power field. HPF: high power field.

Supplementary Figure S5

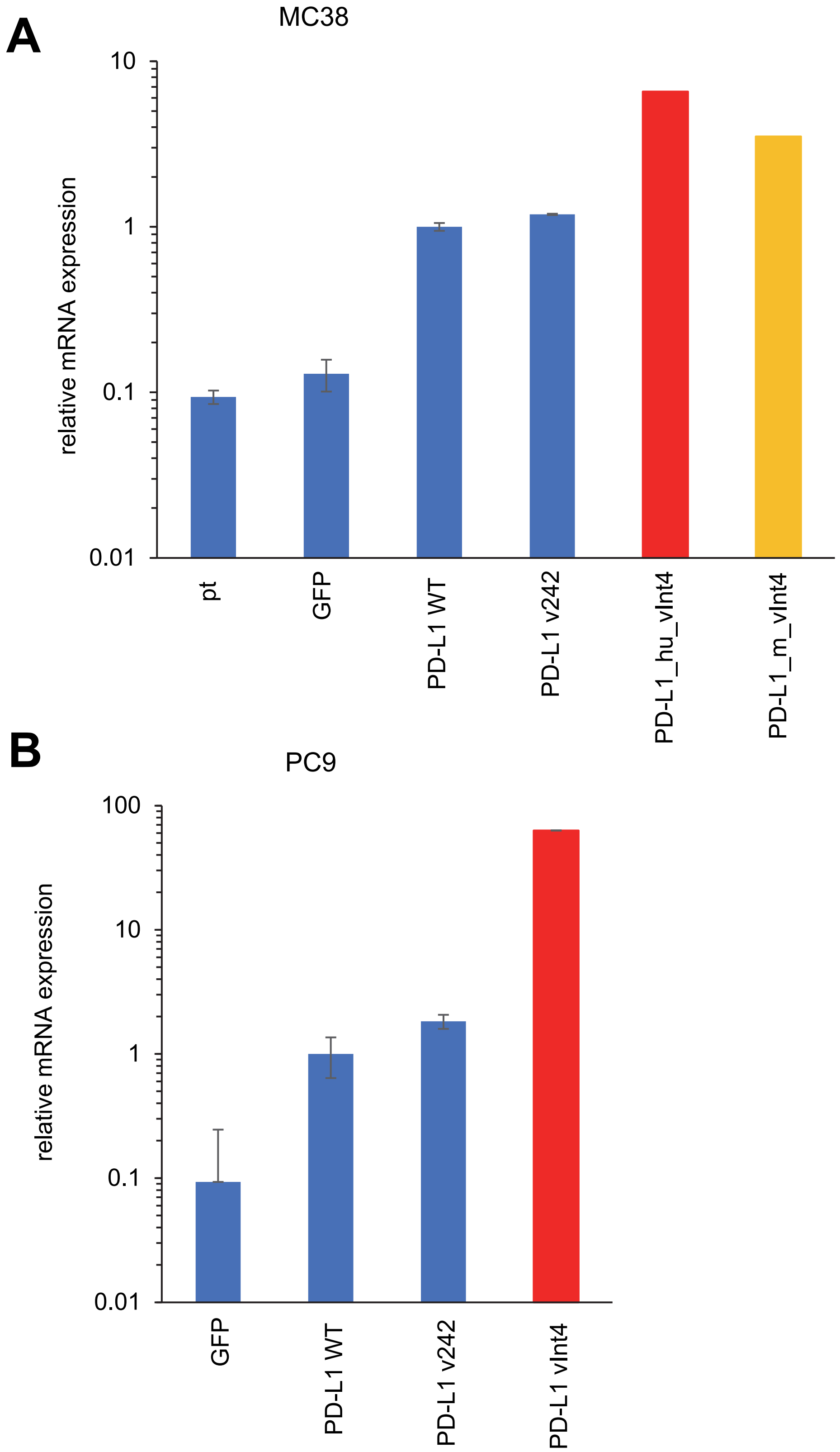

Supplementary Figure S5. Lentivirus-infected MC38 or PC9 cells stably overexpressed PD-L1-vInt4. (A) PD-L1 mRNA levels in MC38 parental cells and splicing variants or GFP overexpressing MC38 cells were quantified with real time PCR. (B) PD-L1 mRNA levels in splicing variants or GFP overexpressing PC9 cells were quantified with real time PCR. Data represent mean ± SEM.

Supplementary Figure S6

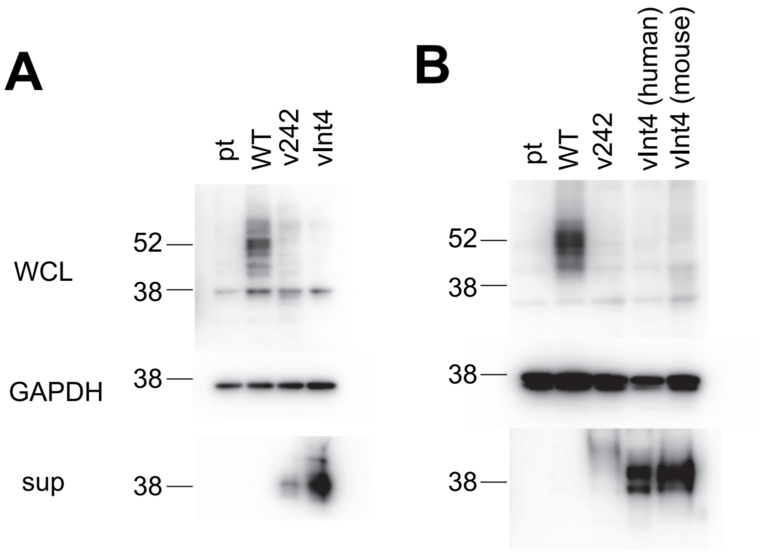

Supplementary Figure S6. Lentivirus-infected MC38 or PC9 cells stably expressed and secreted PD-L1-vInt4 protein into the culture medium. Expression of PD-L1 WT and splicing variants were evaluated by Western blotting for PC9 cells (A) and MC38 cells (B). pt: parental cells. WCL: whole cell lysates.

Supplementary Figure S7

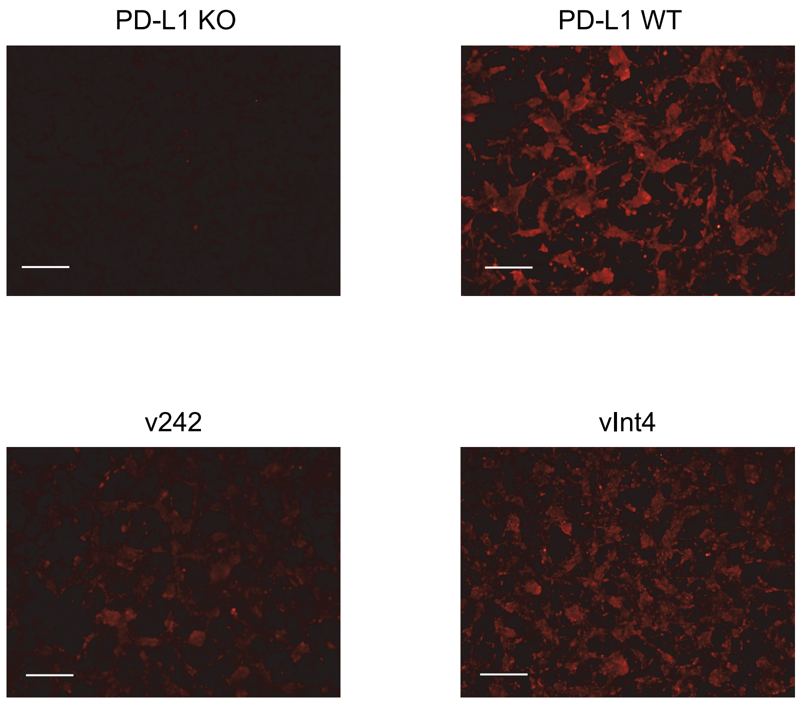

Supplementary Figure S7. PD-L1 expression was confirmed in MC38 cells. Immunofluorescence staining of murine PD-L1 and its variants, using mouse PD-L1 antibody (AF1019) and Alexa Fluor 594 labeled rabbit anti-goat IgG as the primary and the secondary antibodies, respectively. The exposure time was 350 msec. Each scale bar displays a length of 100 μm.

Supplementary Figure S8

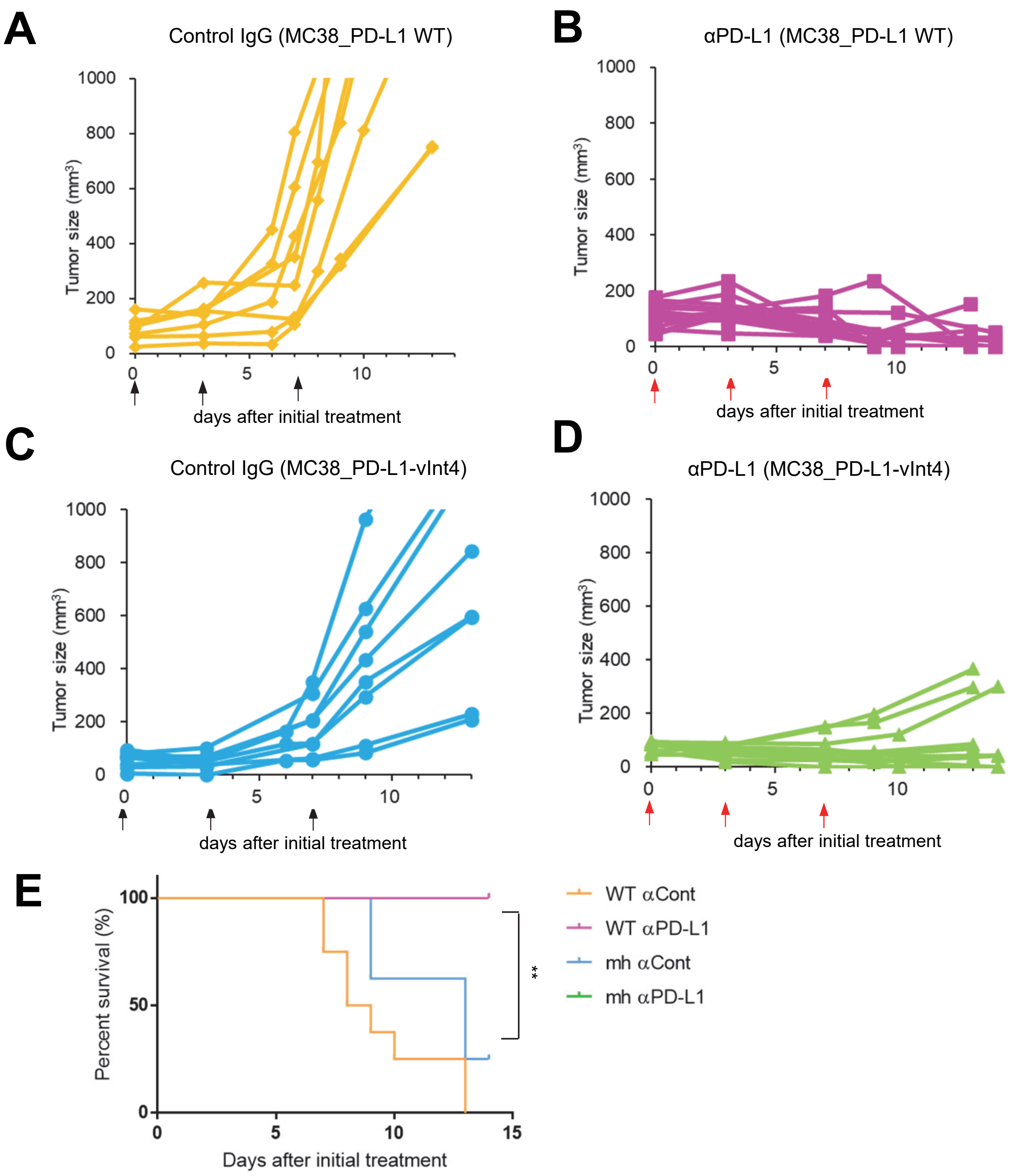

Supplementary Figure S8. Tumor proliferation was suppressed when the dose of αPD-L1 was increased. **(A)(B)(C)(D)** Mice were injected with 5 × 10^5 MC38 cells with overexpression of WT PD-L1 or PD-L1-vInt4 and treated with 200 μg of anti-PD-L1 or control IgG three times a week by i.p. injection. Each figure represents a mice group of: (A) MC38_mPD-L1 WT, treated with control IgG, n = 8, (B) MC38_mPD-L1 WT, treated with αPD-L1, n = 13, (C) MC38_mPD-L1-vInt4, treated with control IgG, n = 8, (D) MC38_mPD-L1-vInt4, treated with αPD-L1, n = 12. The regimen of treatment is indicated by black arrows for control IgG and red arrows for αPD-L1.(E) Kaplan–Meier curves of treated mouse groups in (A)–(D). **, $p < 0.01$ by Gehan-Breslow-Wilcoxon test.

Supplementary Figure S9

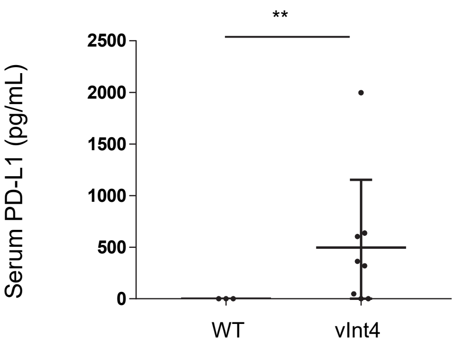

Supplementary Figure S9. Serum PD-L1 levels were compatible with humans'. Serum PD-L1 in mice were measured at day14. Data represent mean ± SEM. **, p < 0.01 by paired two-tail Student's t test.

Supplementary Figure S10

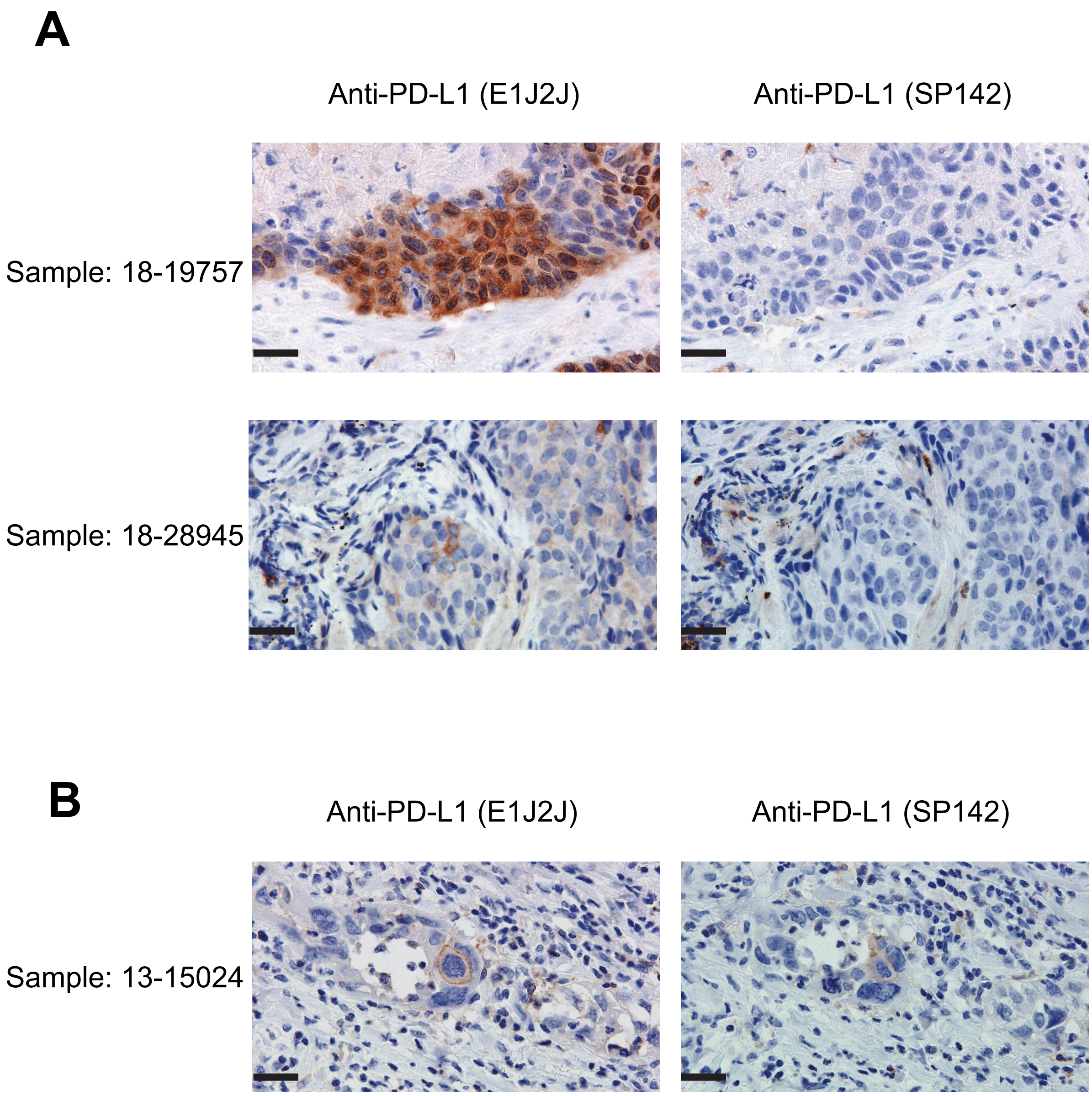

Supplementary Figure S10. PD-L1 staining performed in prior-treatment clinical samples of patients who received atezolizumab for their treatment. (A) Staining pattern of progressive disease cases to anti-PD-L1 blockade therapy. (B) Staining pattern of partial response case. Each scale bar displays a length of 50 μm.
